# Gardens as Science Learning Contexts Across Educational Stages: Learning Assessment Based on Students’ Graphic Representations

**DOI:** 10.3389/fpsyg.2020.02226

**Published:** 2020-09-01

**Authors:** Marcia Eugenio-Gozalbo, Lourdes Aragón, Inés Ortega-Cubero

**Affiliations:** ^1^Department of Didactics of Experimental, Social, and Mathematical Sciences, Faculty of Education of Soria, University of Valladolid, Soria, Spain; ^2^Department of Didactics, Faculty of Education Sciences, University of Cádiz, Cádiz, Spain; ^3^Department of Art Education, Faculty of Education of Soria, University of Valladolid, Soria, Spain

**Keywords:** garden-based learning, science teaching, educational gardens, graphic representations, context-based science learning

## Abstract

The educational use of daily-life contexts is considered a valuable strategy to promote meaningful science learning, since it facilitates the establishment of connections between previous knowledge, personal interests, and new learning. The aim of this work is to provide evidence to support the presence of gardens at educational centers, by assessing key science topics whose learning is promoted at the pre-school, primary, secondary, and university stages. To this end, we analyzed the paired graphic representations of “a garden” that students drew both before and after their participation in a garden-based learning program. Firstly, we obtained the frequency of appearance of every represented element, and afterward characterized the level of change between paired graphic representations. Sample size was of 24–19–25–29 pairs per stage, respectively. Across all stages, an overall improvement in students’ graphic expression was observed, which can be attributed to their experience in the space. At the pre-school stage, the garden favored the establishment of some simple cause-effect relationships which were consolidated at the primary stage, and provided a climate of motivation and affectivity that was evident in the final drawings, given the enormous quantity of details represented, the level of the finished product, and the careful combination and variety of colors. The presence of elements related to water notably increased in final graphic representations from pre-school, primary, and secondary education, thus evidencing that the use of gardens facilitates an approach to responsible water management. At the university stage, students initially demonstrated good knowledge of conventional agriculture, while the gardening experience -which was based on permaculture practices- helped evolve their ideas toward an alternative model of cultivation. The most prevalent science learning across all stages was related to plant knowledge, particularly to their anatomical traits and diversity. Finally, the role of educational gardens as models for students was evidenced, which suggests the importance of teachers and institutions carefully considering which model to offer. Overall, our results support the legitimacy of incorporating gardens to educational centers, particularly for promoting contact with live plants and plant knowledge, and potentially for promoting contact with garden fauna and activities oriented toward learning about it.

## Introduction

The last 10 years have seen a clear decline in the number of young Europeans studying science, which is especially worrying in the context of today’s societies, in which science and technology play an essential role (Rocard et al., 2007). The Rocard report warns that “the origins of this situation can be found in the way science is taught,” and recommends implementing renewed science teaching methodologies based on inquiry (*inquiry-based science education*, IBSE) to promote students’ interest in science (Rocard et al., 2007). In this regard, some studies underline the importance of factors such as teachers’ influence – either positive or negative, teachers’ didactic model, or parents’ income level and education (Hacieminoglu, 2016). In Spain, abundant research has been conducted on the lack of interest in science studies (Solbes et al., 2007; Rodríguez et al., 2011; Solbes, 2011; Robles et al., 2015). Overall, it has great implications regarding the training of future citizens who are committed and capable of facing techno-scientific and social issues (Jocz et al., 2014), which in turn constitutes one of the main aims of current science education, beyond the purely propaedeutic (Acevedo, 2004). An outstanding difficulty lies in the distance between science curricula and daily life: making sense of what they are taught is often very hard for students (Blanco et al., 2012).

The overarching framework of constructivism includes different approaches to science teaching which respond to different visions of science education (Pozo, 1997). One of such approaches is Science, Technology, Society, and Environment (STSE), which incorporates socio-scientific issues when dealing with scientific content (Martínez Pérez et al., 2007; España and Prieto, 2010) and is considered by some to be an appropriate paradigm for selecting the most significant and useful basic content for curricula, under the idea of inclusive science for all (Bennássar et al., 2010). STSE encompasses, among others, teaching strategies based on contextualizing science, which refers “relating it to students’ daily life and showing its interest for personal, professional and social aspects of their future lives” (Caamaño, 2011, p. 21). It was Dewey who introduced the notion *situation*, and considered that the way of experiencing and judging objects or events does not occur in isolation, but as the relationship with a contextual whole (Dewey, 1938, cited in Zapata, 2016). Contextualization, as a strategy, includes both scientific concepts being used to interpret and explain the context, and concepts and models being introduced and developed from a context (Caamaño, 2011). Moreover, it can be combined with other science teaching strategies such as modeling or inquiry (Blanco et al., 2012; Lupión and Prieto, 2014). Blanco et al. (2015) state that a suitable context needs to meet two specific requirements: being relevant to students’ personal, social, and overall daily life, and providing chances to build key science ideas, relationships between them and to theoretical models.

Gardens thus emerge as valuable contexts for science teaching that arouse students’ interest and motivation toward learning (Eugenio-Gozalbo et al., 2019), help connect abstract learning with individual and collective experiential learnings, and integrate activities of daily life with curricular content (Tello and Díaz, 2017). A review of the impact of garden-based learning on academic outcomes revealed consistent positive results across programs, students samples, and school types (Williams and Dixon, 2013). Interestingly, gardens are also useful tools to engage students with food production and help reflect on production and consumption models (Llerena and Espinet, 2017; Pineda Encalada and Estrada Martínez, 2019), and promote healthy eating habits (Berezowitz et al., 2015; Davis et al., 2015; Ohly et al., 2016). They moreover allow students gaining outdoor experiences, which is valuable since a growing body of empirical evidence exists on the fact that outdoor classrooms increase wellbeing and boost subsequent classroom engagement (Kuo et al., 2018; Largo-Wight et al., 2018), and on the impacts of greening schoolyards on children’s health and wellbeing (Kelz et al., 2013; Van Dijk-Wesselius et al., 2018). Finally, they are versatile tools that can be used throughout all educational stages: pre-school education (Murakami et al., 2018; McMillen et al., 2019), primary education (Nury et al., 2017; Dyg and Wistoft, 2018), secondary education (Ruiz-Gallardo et al., 2013; Eugenio et al., 2017), and higher education (LaCharite, 2016; Eugenio-Gozalbo et al., 2020).

The aim of this work was to provide evidence on the usefulness of gardens as contexts for science teaching across various educational stages, for which we draw on the analysis of students’ graphic representations in order to assess their mental representations of scientific contents. Thus, the main research questions posed are:

(a) Do gardens act as real-life contexts that foster scientific learning?
(b) What particular aspects of science learning are promoted by the use of gardens across a range of educational stages?

## Theoretical Framework

Drawing is fundamental for young children as a form of self-affirmation and personal expression (Lowenfeld and Brittain, 1970). Thus, children’s drawings reveal not only their knowledge about the world, but also the emotional relations they establish with things, other people, and living beings (Lowenfeld and Brittain, 1970; Lowenfeld, 1973), while artistic expression is known to contribute to an intensified perception of oneself and the environment (Michael, 1981; Eisner, 2004). All young children draw in a similar way, by using a series of characteristic symbols that are related to biological aspects, as explored by Kellogg (1986) and Stern (2008). More recently, Matthews (2002) delved more deeply into the importance of visual perception and kinesthetic experiences as a stimulus for children to organize lines and shapes in the way they do. Martínez-García (2004) considers that graphic expression integrates the emotion of movement, aesthetic and symbolic factors, producing non-verbalized primal meaning that holds explicit significance when figurative motifs emerge. Overall, it is currently accepted that children’s drawing is a language modeled by certain rules. There is a connection between children’s mental schemes about things (significant knowledge) and the way they draw them. In addition, there are conditioning factors for representation that are strictly related to the formal development of drawing as a language.

Preschoolers draw spontaneously and, in their drawings, they try to reflect the things that are important to them. This explains the personal selection of elements in drawings and the omission of other elements, which may, however, be implicit. Moreover, they represent the space subjectively, for which graphic motifs may be floating all over the paper (Lowenfeld and Brittain, 1970). At this stage, color is not related to reality, in a visual sense, but used for either aesthetic (a certain shade is beautiful) or practical reasons (the crayon is bigger and easier to hold). According to experts, color is also strongly connected to inner emotions (Read, 1996; Machón, 2009). Finally, identifying themselves with different elements of the image constitutes a very usual compositional strategy (just think about the animation of inanimate objects, or the gestures they make while drawing). At primary school, children tend to keep the paper in a fixed position when drawing, which favors the appearance of a baseline that helps them represent the ground convincingly. They will then draw the main motifs of the image perpendicularly to that line (Lowenfeld and Brittain, 1970; Melero, 2004). However, problems appear when children need to reflect a particular flat space “on the ground” (such as a lake). A vegetable garden can be considered one of such problematic spaces, although it can contain some “tall” elements that might help, such as plants, trees, and even human figures. With this in mind, it is reasonable to expect many spatial variations and creative solutions in the way that elementary school children approach the representation of a garden. Finally, the use of different points of view in a single drawing, and the absence of overlapping elements are common at this stage, because children want to reflect very clearly what they know about things, rather than their visual appearance (Luquet, 1972).

At secondary school, the awakening of the self-critical awareness inherent to human adolescence entails a certain inhibition of the creative impulse that results in colder, less expressive graphic representations. Some authors appreciate a decline in the power of self-expression in the transition to this pseudo-naturalistic stage, which is very difficult to avoid without specific training (Eisner, 2004). The human figure is rarely represented, and the combination of different points of view characteristic of children’s drawing still predominates. Attempts to produce a sense of depth can be observed, such as banded composition, linear perspective, or decreased size of objects. Other advances are shadows, which constitute attempts to create volume, and a conscious adjustment of proportions. Drawing frequently takes refuge in stereotypes: graphically defined, easy and accepted formulas to represent certain objects, either own-created or captured from the immediate environment (for instance, from video games, book illustrations, or cartoons), which can be dragged from previous stages (Parini, 2002; Martínez-García, 2004). Finally, most university students show similar characteristics (Fernández-Díaz et al., 2018), since they have rarely had the chance to follow extensive, appropriate art programs aimed at developing their abilities and strategies for graphic representation (Cuenca, 1997; Caeiro et al., 2018).

Drawing connects with the descriptive tradition of the natural sciences, since it involves recording data (Sanders, 2007). Moreover, such data can be used to detect insights and patterns (Katz, 2017). In science teaching, graphic representations are considered a useful tool to, among other things, elicit a student’s personal conceptions of a certain topic before formally addressing it (Ainsworth et al., 2011), thus helping educators take didactic decisions oriented toward improving the teaching-learning process. Giordan and Vecchi (1988) addressed the existence of obstacles in the appropriation of scientific knowledge, and to theoretically define the notion of personal conceptions; their work emphasized the need to elicit them by means of strategies helpful to unveil what is significant for a person, such as drawing. Drawing on graphic expression is a particularly useful strategy in the case of children, since drawing is a meaningful activity (Scheuer et al., 2002) that deeply connects with their personal experience, and allows them to express further significance than just the verbal (Dai, 2017). There exists an extensive corpus of studies that use drawings to detect personal conceptions about scientific topics in students of all ages (Köse, 2008; Margel et al., 2008; Anderson et al., 2014; Ruìz and Palomeque, 2015; Villarroel et al., 2016). In addition, there is a theoretical approach based on the construction of knowledge through drawings (Sanders, 2007; Anderson et al., 2014; Chang, 2017), since graphic representation is considered “an opportunity for the child to construct the science concept” (Chang, 2017), also regarding plant structure and function (Anderson et al., 2014). However, some authors warn of problems that were already assessed in classic treatises, such as “the fact that some students might choose to draw objects that are easy to depict, rather than their immediate associations” (Neumann and Hopf, 2017). Finally, the richness and creative dimension of drawing is also appropriate to reveal affective experiences in nature (Nyberg and Sanders, 2014).

## Methods

### Research Contexts

As university teachers, we became involved in several funded educational research projects focused on the use of gardens, which provided us with the chance to be directly involved in garden-based science programs at the precedent educational stages, an essential condition in order to assure data quality.

#### Pre-school Education

**FIGURE 1:**
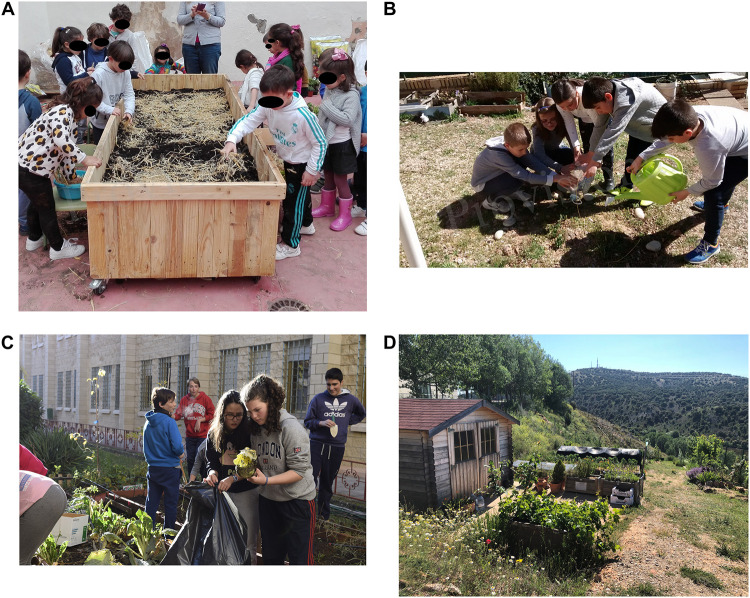
shows the educational gardens at: **(A)** pre-school, **(B)** primary, **(C)** secondary, and **(D)** university. Figures **(B,C)** have been previously published.

A total of 44 4-year-old participated in a research project granted by the regional government of Andalusia (PIV-040/17) and conducted during the 2017/2018 academic year, the main objective of which was to promote the development of scientific competence through using a garden. A raised bed was installed on the cement floor of a courtyard of an approximate extension of 260 m^2^ and which had not previously been home to plant elements (Figure 1A). Students’ participation was continuous throughout the entire project: from design to implementation, maintenance and care, and final consumption of products. In the garden, earthworm humus was used as fertilizer, seeds were organic and seasonal, and straw mulch and crop associations were used. Moreover, reused material such as pallets, cleaning product containers and planters were also used to also create a vertical garden.

#### Primary Education

A total of 40 9-year-old participated in a research project granted by the regional government of Castile and León (EDUJCYL2016-INV03) and conducted during the 2016/2017 academic year, the main objective of which was to assess whether using a garden might improve learning in various disciplines, motivation toward learning, and environmental awareness. There were children from ten different countries at the school, and a fundamental purpose of incorporating the garden was to provide socialization experiences based on values of equity, reciprocity, cooperation, and integration in favor of coexistence. A *garden of the senses*, consisting of an outdoor space limited by metallic fences where cultivation boxes, flowerpots and bags filled with soil were located (Figure 1B), was initiated together with a composting project to recycle students’ home organic waste. Some of the techniques used in the garden were mulching with straw and grass, and using compost as fertilizer. Activities were conducted on different soil types, insects and their habitats, earthworms, seed types and their dispersal, and recognition of tools and horticultural species.

#### Secondary Education

A total of 50 13-year-old students participated in a research project granted by the regional government of Castile and León (EDUJCYL2016-INV03) and conducted during the 2016/2017 academic year, the main objective of which was to assess whether using a garden might improve learning in various disciplines, motivation toward learning, and environmental awareness. At this high school, the garden was used to promote active learning and inquiry-based science learning. The school had a courtyard where three 6 m long × 1 m wide terraces were enabled for cultivation (Figure 1C). A small greenhouse for seedling germination and initial growth, and a drip irrigation system were also available. Activities specifically designed and linked to the official Biology and Applied Sciences curricula were implemented in the garden, with students planning crops and investigating about optimal plant growth conditions, and soil characteristics and composition.

#### Higher Education

A total of 32 pre-service teachers aged 21 years participated in the compulsory subject “Natural Sciences” (Degree in Pre-School Teacher Training, Faculty of Education of Soria, University of Valladolid) during the 2017/2018 academic year, in which science content and its specific didactics for pre-school education were addressed, with a garden being used as the context. The garden consisted of a space of about 350 m^2^, structured in several terraces, where there were various elements: an outdoor table and benches, a tool house, a water storage drum, an insect hotel, a composter and a vermicomposter, several cultivation boxes, two raised beds, and a lateral strip conditioned as a floral band to attract pollinators, with several specimens of fruit trees (Figure 1D). In this garden, agricultural activities follow permaculture principles and techniques, making the most of existing resources such as space, crop association and rotation, straw and grass mulching, and composting and vermicomposting to minimize waste production and to enhance soil fertility, among others. Such techniques involve procedural learnings, which are accompanied by conceptual learnings about matter cycles, limited existence of resources and limits to production, biotic interactions, the role of decomposers, soil ecosystem, etc. The attitudinal learning, which is fundamentally intended, is respect and care for life. Finally, pre-service teachers also learn how to use a garden for educational purposes.

### Data Collection

This work draws on students’ graphic representations of “a garden” (namely, their personal model of what a garden is) before and after the educational intervention. Data collection was conducted similarly across stages, with some particular adaptations to make it more appropriate to students’ cognitive development. In pre-school education, graphic representations were not drawn synchronically by all children, but by one child from each table at a time (they sat in groups of four children), in order to avoid their copying each other. Moreover, the teacher asked them to verbally explain the elements they had represented, and took notes on drawings in order to support graphic analyses. In primary education, the teacher explained the assignment as follows: “Close your eyes. Imagine that it is a great day and that you are watching a garden. It can be a garden you have visited, or a relative’s garden, or a garden that you have seen in a movie… any garden. What do you see?” The teacher also explained that it was not an exam, and there was no correct answer, and finally asked students to draw the garden. In secondary education, students were asked to graphically represent a garden and were given total freedom to do so. Accordingly, the teacher answered “whatever you want” to any questions about what or how to proceed. Similarly, in higher education, students were asked to draw a garden, any garden, preferably in colors, and to identify with words what they were drawing, in order to reduce the chances of misinterpretation. They were explained that this assignment corresponded to data collection on their own conceptions of what a garden is, for which they were completely free, since science misconceptions constituted one of the topics addressed in their subject.

### Data Analyses

Final sample sizes per stage ranged between 19 and 29 paired graphic representations. Sample selection was based on two criteria: (a) that students had participated regularly in classes in the garden, and (b) that graphic representations were unequivocally paired. Thus, for pre-school education, 24 paired samples corresponding to 4 year-old children were considered. For primary education, 19 paired samples corresponding to 9 year-old children were considered. For secondary education, 25 paired samples corresponding to 13 year-old students were considered. And finally, for higher education, 29 paired samples corresponding to 21 year-old students were considered. Such graphic representations were analyzed from two perspectives: Firstly, represented elements were minutely identified and listed, based on a previous work conducted exclusively with secondary education students, which was completed when new elements appeared, and their frequency of appearance (in%) was calculated (see Eugenio et al., 2017). Secondly, paired graphic representations were classified into groups based on the observed level of change or evolution (1 = null or low, 2 = medium, 3 = high or very high). For the categorization of change between paired drawings, clear criteria were used that allowed different researchers to take decisions. In any case, this classification was reviewed by one of the authors, specialized in graphical representations and their evolutionary interpretation. As stated in the theoretical framework, two keys for the interpretations were understanding drawing as a language, and that certain types of representations are linked to determining factors of each graphic stage.

## Results

### Pre-school Education

Table 1 shows the frequency data for the general categories of items described in this subsection.

**TABLE 1:** Frequency of appearance (%) of the different elements of a garden in pre-school and primary education students.

	**Pre-school education**			**Primary education**		
	**Initial GR (%)**	**Final GR (%)**	**Difference**	**Initial GR (%)**	**Final GR (%)**	**Difference**
Isolated garden	75	87	12	84	68	−16
Fenced garden	12	8	−4	37	42	5
Trails within	4	0	−4	0	5	5
Cultivation on the ground	67	62	−5	95	64	−31
Raised beds	0	25	25	0	21	21
Flowerpots	8	0	−8	26	5	−21
Structures	4	0	−4	0	16	16
Sun	17	62	45	21	58	37
Soil	58	87	29	58	89	31
Irrigation elements	29	58	29	37	58	21
Tools	4	8	4	21	37	16
Animals	12	17	5	10	21	11
Humans	17	25	8	16	21	5
Trees	4	8	4	32	32	0
Plants	92	100	8	63	79	16
Seeds	8	46	38	10	0	−10

Initially, most 4 year-old children drew exclusively a garden (75%), without separation elements such as walls or fences (only in 4%), and where cultivation occurred prevalently on the ground (67%). Soil was represented in 58% of total drawings, either by means of a baseline, by locating elements on the bottom support edge, or by representing the sky line. Interestingly, some children highlighted this element as a closed form, more or less longitudinal, that serves as a substrate for plants (12%). Finally, a few children drew a succession of flowerpots (8%). After the educational gardening experience, the general structure remained very similar: 87% of children represented only the garden, rarely with separation elements (8%), and cultivation occurred predominantly on the ground (62%). However, the number of children who represented raised beds as large containers of soil, some of them with wheels (such as those at school) increased to 25%. Notably, soil representation increased to 87%. Finally, the representation of a succession of flowerpots disappeared. Other elements besides the garden -such as a house- were represented in 18 and 8% of drawings before and after, respectively.

Initially, water was scarcely represented (29%), mainly in the form of rain or clouds (in 6 graphic representations, GR hereafter) or watering cans (in 1), and the sun appeared in only 17% of drawings. Tools and trees were practically absent (96% in both cases), people were included only in 17% of cases, and animals in 12% (bees in 1 GR, butterfly in 1 GR, and worm in 1 GR). After the educational gardening experience, notable increases in water representation (58%), mainly through rain and clouds (in 14 GRs) or watering cans (in 2), and in sun representation, occurred (62%). Tools and trees continued to be absent in most drawings (92% in both cases), and animals to be scarcely represented (17%) (worms in 4 GRs and birds in 1 GR). Human figures slightly increased (25%), consisting in representations of children themselves, performing actions such as watering (in 3 GRs), or in a contemplative attitude (in 3).

Most representations (92%) included plants, prevalently corresponding to a graphic formula: the flower (62%), which constitutes a recurring structural element for children at this stage, also for the representation of other elements such as hands, for instance. In several drawings, other products from the garden appeared, such as carrots (in 4 GRs), apples (in 2), peppers (in 2), lettuces (in 1), tomatoes (in 1), and broccoli (in 1), generally laying on the ground (plants were not represented). Seeds appeared in 8% of drawings. After the educational gardening experience, all graphic representations included plants, with flowers or “flower-type” plants still prevalent (in 22 GRs). A greater variety of plants was represented, and more minutely (including parts such as roots, leaves, or fruits): carrots (in 6 GRs), lettuces (in 5), tomatoes (in 4), zucchinis (in 2), sunflowers (in 1), strawberries (in 1), aubergines (in 1), corn (in 1), oranges (in 1), and pears (in 1). Seed representation notably increased (46%).

In paired graphic representations, an overall drastic change was observed, with final drawings being far richer and more interesting. Four paired drawings were classified in the group of null or low change (16.7%). A medium change occurred in 8 paired drawings (33.3%), with final representations being generally richer and more artistic than initial ones, and including more elements but especially a greater level of detail. Finally, 12 paired graphic representations were classified in the group of high or very high change (50%), with final representations characterized by the inclusion of elements such as water, the sun, higher diversity of plants represented with detail, and by higher aesthetic level. An example of each category is included as Supplementary Material.

### Primary Education

Table 1 shows the frequency data for the general categories of items described in this subsection.

Initially, most 9-year-old children drew exclusively a garden (84%), without considering any other elements, whereas the rest (26%) represented a row of flower pots on a baseline. In 37% of graphic representations, the garden was conceived as a space limited exclusively by a line, but without fences or wall-type separation elements, and without trails or roads to move within. Cultivation occurred on the ground (95%), with soil being explicitly represented in 58% of drawings, and furrows or crop lines explicitly included in half of those (42%). After the educational gardening experience, 1/3 of drawings featured other elements, such as a house (in 2 GRs) or a larger building, likely the school building (in 2), and a greenhouse (in 1). The garden continued to be a limited area in 42% and the frequency of appearance of walls or fences increased (16%). Cultivation modes diversified: on the ground (47%), in cultivation boxes (in 21%), flowerpots (5%), or combinations of those elements (27%). Soil was explicitly represented in 89% of drawings; furrows were now much scarcer (11%).

Initially, water was represented in 37% of drawings, either as clouds (in 3 GRs), watering cans (in 2), aquifers (in 1), or lakes (in 1), and the sun was represented in 21% of drawings. Tools were mostly absent (in 80%), human figures were included in only 16% and were represented as gardeners either watering (in 2 gr) or digging (in 2). Such figures were constructed from the stereotypical “stick man,” which is not a natural construction, but influenced by adult drawing. Animals representation was scarce (11%): only birds (in 4 gr), and fishes (in 1). Finally, trees appeared in 1/3 of drawings and were stereotyped (apple type). After the educational gardening experience, notable increases in sun (58%) and water representation (58%) occurred, in this last case by incorporating elements such as watering cans (in 6 GRs), clouds and rain (in 5), buckets (in 1), taps (in 1), and drip irrigation systems (in 1). Likewise, the presence of tools also increased (37%), represented by items such as watering cans (in 6 GRs), shovels (in 3) working tables (in 2), rakes (in 2), gloves (in 1), and baskets (in 1). Human figures were represented even less (21%), consisting of children taking some kind of action, such as watering (in 2 GRs) or working (in 1). Animals continued to be scarce (21%), but their diversity increased: snails (in 2 GRs), cats (in 1), butterflies (in 1), bees (in 1), ladybugs (in 1), and birds (in 1). Finally, trees were equally represented (32%).

Initially, plants were observed in 63% of drawings, predominantly as flowers (in 6 GRs) -leaves and roots appeared in only 1 drawing each– and fruits arranged in a row on the ground, such as tomatoes (in 6 GRs), carrots (in 3), lettuces (in 3), onions (in 3), strawberries (1), and zucchinis (in 1). After the educational gardening experience, the presence of plants increased to 79%, and children prevalently represented vegetables with greater effort, so that they were recognizable. Moreover, a greater diversity of vegetables appeared: carrots (in 8 GRs), tomatoes (in 5), aubergines (in 3), peppers (in 3), pumpkins (in 2), peas (in 2), and lettuces (in 1). The presence of flowers increased (in 11 GRs), and they were represented with more detail: leaves were also drawn (in 6 GRs) in addition to the flower (in 11).

In developmental terms, this group of graphic representations was poorer than those of other levels. Regarding paired graphic representations, 4 pairs (21%) were classified in the group of null or low change, 8 pairs (42.1%) in the group of medium level of change, since they incorporated some elements that improved representations from an aesthetic or quality point of view, and finally, 7 pairs (37%) were classified in the group of high level of change, since significant changes were observed. An example of each category is included as Supplementary Material.

### Secondary Education

Table 2 shows the frequency data for the general categories of items described in this subsection.

**TABLE 2:** Frequency of appearance (%) of the different elements of a garden in secondary and university students.

	**Secondary education**			**Higher education**		
	**Initial GR (%)**	**Final GR (%)**	**Difference**	**Initial GR (%)**	**Final GR (%)**	**Difference**
Isolated garden	72	76	4	64	36	−28
Contextualized garden	20	20	0	32	68	36
Fenced garden	52	60	8	60	28	−32
Trails within	28	56	28	12	60	48
Cultivation on the ground	76	60	−16	100	0	−100
Raised beds	0	40	40	0	52	52
Structures	40	44	4	44	96	52
Sun	36	40	4	16	32	16
Soil	36	40	4	76	92	16
Irrigation elements	28	56	28	84	80	−4
Tools	20	48	28	52	52	0
Animals	16	4	−12	8	28	20
Humans	8	0	−8	4	12	8
Trees	64	60	−4	52	72	20

Initially, students basically represented an isolated garden (72%), consisting of a space delimited by fences (51%) where cultivation occurred on the ground (76%). Strikingly, some graphic representations consisted of a “compartmentalized space” (in 1 GR) or a “collection of plant fruits” (in 2 GRs); they did not represent a real garden. After the educational gardening experience, students continued to represent mostly an isolated garden (76%) limited by walls or fences (60%), where there were paths to move on (56%). While a model of cultivation on the ground continued to predominate (60%), cultivation in raised beds was also represented (40%).

Initially, elements related to irrigation and working tools were poorly represented (28 and 20%, respectively), although structures such as benches, tables, or greenhouses appeared often (40%). Regarding living beings, people and animals were practically absent (only in 2 and 4 GRs, respectively), and, of the latter, only birds were represented. Trees appeared in numerous graphic representations (64%), and were always stereotyped, with “the apple tree” being the most frequently represented tree species (in 5 GRs). After the educational gardening experience, the frequency of appearance of irrigation and working tools increased to 56 and 48%, respectively. Structures were similarly represented (44%), but with greenhouses and composters prevailing over others. Regarding living beings, people and animals continued to be practically absent (in 0 and 1 GR, respectively). Graphic patters of tree representation remained similar.

Initially, plants were all stereotyped, or represented by symbols or even by their written name (in 3 GRs). Tomato plants were the most frequently represented crop (64%), and plant anatomical knowledge varied from very low (fruit on the ground, in 1 GR), to medium (a “stick with pendulous fruits” or “a bush with fruits,” in 8 GR), and finally high (a complete plant where stem, branches, leaves, and fruits are distinguished, in 6 GR). It was followed by carrots (48%), represented either below ground (in 8 GRs) or above ground (in 4). Pumpkins were the third most frequently represented crop (28%): 4 students drew only the fruit on the ground, and 3 drew a complete plant. After the educational experience gardening, plants continued to be stereotyped or represented by symbols in 2 GRs. Carrots became the most common crops (72%), mainly but not always below ground (in 13 vs. 5 GRs). These were followed by onions and tomato plants (60% each). Onions appeared below ground in 9 cases, and above ground in 6. Tomato plants were represented as fruits on the ground (in 5 GRs), as “a stick with pendulous fruits” or “a bush with fruits” (in 6 GR), and as a complete plant (in 3). The representation of lettuces increased, from 52 to 64%, and that of strawberry beds, from 8 to 28%.

Regarding paired graphic representations, 6 pairs (24%) were classified in the group of null or low change, since the structure of the gardens and the items represented were approximately the same in the initial and final graphic representations, with very small differences, such as the inclusion of a new element. Eight pairs (36%) were classified in the group of medium change, since several new, important elements were included in the final drawing, although overall structure and context of the garden was very similar in both representations. Finally, 11 pairs (40%) were classified in the group of high or very high change; in such cases, several new, important elements were included in the final graphic representation, together with changes in garden structure and contextualization. An example of each category is included as Supplementary Material.

### Higher Education

Table 2 shows the frequency data for the general categories of items described in this subsection.

Initially, students represented an isolated garden (64%), where cultivation occurred on the ground (100%) and furrows frequently appeared (62%). Paths to move on were present in 40% of graphic representations. After the educational gardening experience, most students represented a garden contextualized in a landscape (68%). Raised beds for cultivation appeared in half of the graphic representations (52%), along with cultivation on the ground. Within the garden, both paths (60%), and empty space appeared (48%); and tiles -such as those in the university garden- were represented on raised beds to walk along them without compacting soils (36%).

Initially, elements related to irrigation were abundant (84%), including wells or fountains (in 6 GRs), hoses (in 6), drip irrigation systems (in 6), drums of water (in 5), or rivers (in 5). Noticeably, 8 students represented more than one of these elements. Tools appeared only in 52% of graphic representations: mainly shovels (in 10 GRs), hoes (in 9) and rakes (in 5). Five students represented “compost,” 2 “herbicides,” and another 3 trailers or plow tractors; only 1 represented a scarecrow and another a “bag to scare birds.” As for other elements, warehouses or sheds for tools (in 5 GRs) appeared, and also 1 greenhouse and 1 composter. After the educational gardening experience, irrigation elements remained abundant (80%), and 13 students represented more than one. Tools were similarly represented (52%), but their diversity increased to include wheelbarrows (in 6 GRs), shovels (in 3), boots and gloves (in 1), together with shovels (in 8 GRs), hoes (in 7) and rakes (in 5). Three students represented “compost,” but none represented “herbicides”; plow tractors and scarecrows also disappeared. Structures were profusely represented (96%): warehouses/tool sheds (in 20 GRs), composters (in 12), vermicomposters (in 5), culture boxes (in 4), insect hotels (in 3), and banks (in 3).

Initially, fruit trees were represented in 52% of graphic representations, and were stereotyped in all but one case (a plum tree that was represented with some anatomical detail). The most commonly represented tree species were apple and lemon trees (in 4 GRs each). Fauna was absent: one student represented chickens, another stereotyped birds, and a third, ants. Similarly, human figures appeared in only 1 graphic representation: two gardeners, a male and a female. After the educational gardening experience, fruit trees were represented in 72% of cases, and were always stereotyped. The diversity of tree species increased to include apple (in 6 GRs), cherry (in 3), almond (in 2), orange (in 2), pear (in 1), and lemon (in 1) trees. Fauna appeared in 28% of cases, consisting mainly of stereotyped birds (in 4 GRs), flies (in 1), earthworms (in 1), snails (in 1), and butterflies (in 1). Human figures appeared in only 3 final graphic representations (12%).

Initially, the most commonly represented elements were vegetable crops, for which a close anatomical knowledge was evidenced, although not always detailed. The most represented crops were tomato plants (in 21 GRs), lettuces (in 19), onions (in 16), and carrots (in 15). Tomato plants were always represented as a plant (never an isolated fruit on the ground), and, in 48% of the cases, staked. Carrots and onions were mostly represented underground, with only 1 and 2 exceptions, respectively. Potatoes appeared in 40% of graphic representations: from the word “potato” (in 4 GRs), to isolated potatoes either on (in 1) or below ground (in 3), and a complete plant (in 2). Some characteristic crops of students’ areas of origin were outstanding: borage (in 1 gr), edible thistle (in 1), vineyards (in 2), or beet (in 1). Finally, a student left a piece of fallow land (and wrote “uncultivated”). After the experience at the university garden, crop associations and companion plants appeared in 40 and 52% of the graphic representations, respectively. Tomato plants (in 20 GRs), lettuces (in 20), onions (in 16), and carrots (in 12) continued to be the most represented. Garlic (in 10) and spinach (in 6) appeared, whereas potatoes declined in importance (in 3). Among companion plants, mainly basil (in 7 gr), “flowers” (in 5), rosemary (in 4) and lavender (in 4) were represented, but also sage (in 2), peppermint (in 2), thyme (in 1), and calendula (in 1) were included. In one graphic representation, compost was located at the base of plants’ stem.

Regarding paired graphic representations, 6 pairs (24%) were classified in the group of null or very low change, 9 pairs (36%) in the group of medium change, and 10 pairs (40%) in the group of high or very high change. An example of each category is included as Supplementary Material.

Table 3 shows the level of change of identified elements across educational stages.

**TABLE 3:** Changes in frequency of appearance (%) of the different elements represented in drawings, across educational stages.

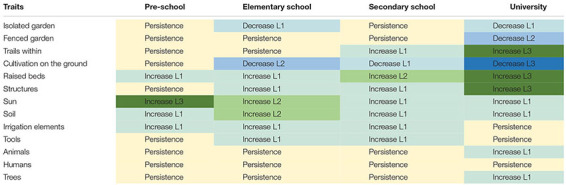

*Changes are calculated as (final-initial). Persistence (cream) = changes under 14%. Increases (green) and decreases (blue) are classified in 3 levels: L1: 15–29, L2: 30–44, and L3: ≥45.*

## Discussion

This work aimed to assess students’ learning progress related to the use of gardens as contexts for science teaching from pre-school to higher education. We based our research on drawings of a garden, a flat space on the ground whose representation involved difficulties that students solved by using a range of strategies, such as selecting the most revealing point of view, or the easiest to draw, or representing the garden from a combination of different points of view, or even by including explanatory texts. Noticeably, graphic expression is partly dependent on the cognitive maturity of students in a certain age range (Freinet, 1970; Lowenfeld and Brittain, 1970; Luquet, 1972; Matthews, 2002; Martínez-García, 2004; Machón, 2009). The absence of human figures in graphic representations from secondary and university students constitutes an example: when a person is not specifically trained, they continue to represent people as they did as a child. Thus, most adolescents are not capable of representing people with a quality drawing, and this appears as “a feeling of guilt or shame” (Lowenfeld and Brittain, 1970, p. 301). Therefore, there are not representations of the garden as a space for coexistence or socialization from these stages. Moreover, graphic expression is promoted by the observation of reality, and thus, encouraging children to directly experience space in different ways and have physical contact with things is very important for their natural development (Lowenfeld and Brittain, 1970; Matthews, 2002; Martínez-García, 2004). In our study, and broadly speaking, the quality of students’ garden representations was found to increase across all stages after their experience in learning gardens. Furthermore, it is very common to find stereotypes: trailing symbols from earlier graphic stages which remain through time because there has been no genuine observation of the motif represented; a clear example is given by the representation of trees and birds. Stereotypes, rather than rich and reliable depictions, pursue communicative efficiency: objects are represented “according to economic principles that lead the children to draw them separately and always repeat them in the same way” (Parini, 2002, p. 169).

Fortunately, access to outdoor spaces is one of the most effective ways to enrich the graphic expression of students of all ages, as it facilitates not only direct observation, but also enjoyment of the beauty of nature. This is a classical pedagogical strategy of art teaching, which emerged in the 19th century and still constitutes a desirable standard: “Today there is a lot of literature dedicated to the landscape as an aesthetic experience derived from the observation and experimentation of the physical act of walking” (Marco-Mallent, 2016). Afterward, such feelings of emotional well-being would be revived in the classroom through drawing. Recent studies show positive impacts of outdoor classrooms on students’ well-being and subsequent engagement (Kuo et al., 2018, 2019; Largo-Wight et al., 2018), and thus, of schoolyard greening on students’ attention, well-being, and health (Kelz et al., 2013; Van Dijk-Wesselius et al., 2018), which links to the use of gardens as learning contexts (Dyg and Wistoft, 2018). In our study, pre-school children’s enjoyable experiences at the garden were reflected in their final drawings by the enormous quantity of represented details, the level of finish, the careful combination and variety of selected colors. The relation between objects’ real colors and their representation is fanciful at this age, and the selection of different and abundant tones involves a love for what the child is drawing and also their deep identification with those elements. It is known that: “What the child draws is always a projective image of his body.” Representing a human being, a house, a ship, a tree, an animal, what he means is “I” (Stern, 1961, 50). Enjoyment and emotional well-being were not so evident in graphic representations from primary students, which were overall less rich, and of lower quality. Lastly, the awakening of the self-critical awareness that occurs in adolescence entails a certain inhibition of the creative impulse that results in colder, less expressive graphic representations (Eisner, 2004). Thus, drawings from secondary and university students were much more rational, with colors adjusted to reality, usually without nuances, and these aspects could not be so clearly perceived.

The concept of what “a garden is” evolved and diversified in all educational stages, from a prevalent initial idea of cultivating on the ground (or in flowerpots, in the case of some pre-school children), to a final idea that included cultivation in raised beds and cultivation boxes, as was experienced in the learning gardens. This is significant, since it opens a vast array of creative possibilities regarding the relation with plants and the manners in which they can be placed near us in our daily lives (Eugenio-Gozalbo et al., 2020). Overall, the diversification of the initially included elements of each type was an outstanding trait of final graphic representations across educational levels: the initial ideas that students held not only about modes of cultivation, but also about watering, tools, structures, crops, tree species, or animals were enriched after the educational gardening experience. Thus, these results confirm what we already observed in a previous work: learning gardens constitute models that greatly contribute to the notion of “what a garden is” that students reconstruct (Eugenio et al., 2017). Changes in paired graphic representations were medium (new and relevant elements were included) or high (moreover, there were also changes in garden’s structure and contextualization) in at least 3/4 of cases at every educational stage: generally, such changes resulted in original ideas of “a garden” becoming more similar “to the garden at my school.” Consequently, it is important for educational institutions and teachers to invest time in deciding the elements they want to include and how they want to cultivate their gardens, since these are key aspects which will influence students’ learning. In the particular case of the university, the contextualized learning experience (Caamaño, 2011; Tello and Díaz, 2017) made students evolve from an initial idea of gardening that was related to conventional agriculture (represented by elements such as “herbicides” and trailers or plow tractors) to a final idea that was closer to organic agriculture (composters, insect hotels, associations, and companion plants), which in turn is associated with key learning in the fields of environmental and food education (Eugenio-Gozalbo et al., under revision).

Regarding scientific learning, significant progress was observed in the 4-year-olds. After the educational gardening experience, some elements and processes were represented which involve the establishment of simple cause-effect relations, such as: “plants come out of seeds,” “plants must be watered,” or “plants need the sun.” Meaningful learning occurs when “new knowledge is meaningful for individuals, and prior knowledge acquires new meanings or greater cognitive stability” (Moreira, 2012, p. 30), which was the case. Moreover, content learned at early ages may remain as children grow, particularly when it is reinforced across educational stages and if children have the chance to actively construct such content (Witt and Kimple, 2008), with indicates that the convenience of using gardens as learning contexts across subsequent educational stages. Consistently, elements such as water and the sun were incorporated into the final drawings of the 9-year-olds, thus reflecting that they handled more complex cause-effect relationships. Overall, an increased frequency of representation of elements related to irrigation was observed from pre-school to secondary education, thus indicating that students developed a growing awareness of the importance of water and what gardens offer in a real-life context that facilitates addressing responsible water management (Blanco et al., 2015). Accordingly, a project consisting of a collaborative technological design of an irrigation system for a school garden was used with the objective of promoting the discursive construction of the eco-citizen competence of primary school students (Rekondo et al., 2015).

Two elements can be underlined as the most prevalent scientific learning across stages: firstly, the development of observation, which is considered a basic scientific skill, and secondly, the improvement of anatomical plant knowledge. Thus, the 4-year-olds were able to overcome the “flower-type plant” -a motif that was expected, since it constitutes a well-known basic element in spontaneous young children’s drawing development (Kellogg, 1986; Martínez-García, 2004; Stern, 2008), finally employing a higher level of detail and concreteness when representing plants, including a greater diversity of plants, and using some basic scientific vocabulary (“stem,” “leaves,” and “roots”). In turn, the 9-year-olds represented plants more profusely, with a higher level of detail and with efforts to make them individual and identifiable; they included a greater variety of crops. In the 13-year-old students, the most commonly represented crop species changed to include those that had been cultivated at the learning garden, and the progress in anatomical plant knowledge was shown by shifts from representing only fruits -for instance, in the case of tomatoes or pumpkins- or tubers -for instance, in the case of potatoes- on the ground, toward representing a whole plant structure with stem, branches, and leaves, where fruits or tubers were properly positioned, or by representing some vegetables such as onions or carrots below ground instead of on the ground. Similarly, in the 21-year-old students, both crops and tree types moved at least partly toward those that were cultivated at the university garden, and a progress in anatomical plant knowledge was observed.

Contributing to improved plant knowledge is undoubtedly significant. Some 20 years ago, two biology teachers coined the term “plant blindness” (Wandersee and Schussler, 1999) to refer to the fact that humans tend to “not notice” plants in their environments, which they considered to be due to a perceptual inability (Wandersee and Schussler, 2001). Plants, however, play a fundamental role for life on planet Earth, both quantitatively (Bar-On et al., 2018) and functionally (Hiscock et al., 2019), in that they provide humans with numerous goods and services, and they have a great significance in cultural identities (Pardo de Santayana et al., 2014). However, a growing ignorance of plants and an underestimation of their importance is generally detected (Bebbington, 2005), which can be attributed to several factors: while people living in societies where plants are essential for their survival know them closely (Hall, 2011), more than half the world’s population is currently urban (World Watch Institute, 2007) and has thus grown away from them. Moreover, plants are underrepresented in curricula and biology books show a clear zoocentric focus (Schussler et al., 2010; Rodrìguez Miranda et al., 2014). Finally, scientific education approaches “plantness” (Darley, 1990) in inadequate and discouraging manners (Wandersee and Schussler, 2001). Firstly, plants are described by presenting what they “don’t do” (Eugenio, unpublished), and secondly, teaching about plants by using experimental plant material constitutes a challenge for many biology teachers (Tomkins and Tunnicliffe, 2007). However, the extinction of plant species is already extensive (Willis, 2017; IPBES Global Assessment, 2019), and increasing knowledge of them is essential in the work of the scientific education of future citizens (Fančovičová and Prokop, 2011; Suárez-López and Eugenio-Gozalbo, in press).

Our findings support the use of gardens as real-life learning contexts that promote direct contact with living plants and generate important, meaningful learning on this group of living beings. Several conditions are accomplished that legitimize gardens’ incorporation in school yards, that is, in formal education, across all its stages. Firstly, Wandersee and Schussler (2001, p. 6) underlined that “early experiences in growing plants under the guidance of a knowledgeable and friendly adult was a good predictor of later attention to, interest in, and scientific understanding of plants,” which grounds their use at pre-school and primary education. Other experts in the field consider that “affective experiences, through personal encounters, observations and guided explorations, can enhance students’ attention” to plants (Nyberg and Sanders, 2014, p. 142), and that “educational experiences where the ecological and social significance of plants is the main focus are crucial tools to help us to overcome “plant blindness” and challenge “zoocentric” views” (Nyberg et al., 2019, p. 212). Attention has been paid in the field to the use of botanical gardens for this purpose (Sanders, 2007), and it seems evident that gardens can be incorporated into schools, offering regular experiences of contact, growing, observation, caring, and experimentation with plants, and, particularly, with plants that are significant to us, as they provide us with food. It is important to reflect on what strategies to use: for instance, to use students’ interests (Lampert et al., 2019) to trigger affective and meaningful experiences. Drawing may be incorporated both to promote the construction of scientific concepts and to assess them (Eisner, 2004; Sanders, 2007; Anderson et al., 2014).

However, little scientific learning on animals occurred in the study. Interestingly, and despite the number of animal species represented increasing after the educational gardening experience in students from primary to university stages, animals appeared in only a small percentage of total graphic representations. Garden fauna can be abundant, depending on both the biophysical context where the garden is located and on land management -importantly, whether biocides are used or no- (Martínez-Zaporta, 2016), for which gardens can provide useful opportunities to learn about animals, particularly insects. The observed lack of graphic representation of animals is partly due to the scope of the didactic interventions, which did not focus on introducing students to garden fauna. However, and importantly, this points to the need to promote fauna in learning gardens, by means, for instance, of installing insect hotels or melliferous plants that attract pollinators (Bradbury, 2019; Ashton and Ashton, 2020). In addition, it would be useful to implement activities that increase students’ awareness of animal biodiversity, and particularly, of the presence, roles and importance of insects in ecosystems, which may improve both students’ knowledge and behavior toward insects (Eugenio-Gozalbo and Ortega, in prep.; Eugenio-Gozalbo et al., under revision).

Regarding the main limitations of this research, the first one is small size samples, particularly in primary education. This was due to the difficulty, as university teachers, of getting directly involved with reliable science programs contextualized in gardens in the precedent educational stages, which we achieved by pursuing two funded regional research projects: this seemed to us an essential condition to assure data quality. Secondly, we based exclusively on the analyses of graphical representations, which may constitute a limitation, since there are students who express themselves better verbally, and therefore, the fact that a particular element does not appear would not unequivocally mean that they do not know that it is present. In words of Neumann and Hopf (2017): “One of the drawbacks of utilizing drawings, however, is the fact that some students might choose to draw objects that are easy to depict, rather than their immediate associations” (Ibid., 113). For further study on this topic, it is recommended to combine images with text (Coleman et al., 2011) or, to allow older students to use connectors to symbolize processes that provide more information, which was in fact done for university students, but was not analyzed for this work.

## Conclusions

Gardens are appropriate real-life contexts for science teaching across educational stages, where students experience the space and develop observation skills, which is linked to both the development of their scientific knowledge and the evolution of their drawing maturity. In the earlier stages, pre-school and primary education, gardens provided a climate of affectivity and motivation, promoted the establishment and consolidation of simple cause-effect relationships, and developed children’s artistic expression. In the later stages, secondary and university education, the educational experience in gardens involved enrichment and diversification in all the elements present (modes of cultivation, irrigation elements, tools, crops, trees, and animals), thus significantly developing students’ knowledge.

Water emerged as an important issue from pre-school to secondary education, and thus responsible water consumption and water management constitute key environmental topics that can be properly addressed from gardens. The most prevalent science learning across all stages was related to plant knowledge, both regarding anatomical traits and diversity of species. Given its importance, we propose learning gardens as real-life learning contexts from which to promote direct contact and learning on culturally significant, living plants. In this regard, art practices can be useful: graphic representations, and their use in conversations among students to share ideas is recommended.

Finally, and also across all stages, learning gardens constituted models that greatly influenced students’ final idea of “what a garden is,” which has important didactic implications for schools and teachers regarding their choices on what to have and what to do in learning gardens. This includes, on the one hand, the need to implement sustainable agricultural practices, and, on the other hand, the need to promote garden fauna - by means, for instance, of installing insect hotels or melliferous plants that attract pollinators – and to implement activities that increase students’ awareness of animal biodiversity, and particularly, of the presence, roles and importance of insects in ecosystems.

## Data Availability Statement

The raw data supporting the conclusions of this article will be made available by the authors, without undue reservation, to any qualified researcher.

## Ethics Statement

Ethical review and approval was not required for the study on human participants in accordance with the local legislation and institutional requirements. Written informed consent to participate in this study was provided by the participants’ legal guardian/next of kin. Written informed consent was obtained from the minor(s)’ legal guardian/next of kin for the publication of any potentially identifiable images or data included in this article.

## Author Contributions

ME-G and IO-C had previously worked together on secondary students’ graphical representations of gardens. ME-G developed the idea of analyzing a sequence of graphic representations across educational stages. LA contributed with pre-school data. ME-G contributed with primary, secondary, and university data. Data analysis was conducted by the ME-G, LA, and IO-C (supervised by IO-C, a specialist in the area of graphic analysis). The manuscript was written by the ME-G, LA, and IO-C (and coordinated and supervised by ME-G). All authors contributed to the article and approved the submitted version.

## Conflict of Interest

The authors declare that the research was conducted in the absence of any commercial or financial relationships that could be construed as a potential conflict of interest.
