# Supplementary material for: Gardens as Science Learning Contexts Across Educational Stages: Learning Assessment Based on Students’ Graphic Representations
Source: Front Psychol. 2020 Sep 1;11:2226. doi: 10.3389/fpsyg.2020.02226 (PMC7495090; doi:10.3389/fpsyg.2020.02226)
Supplement: Supplementary file 1 [file Data_Sheet_1.PDF]

Supplementary Material

Supplementary Material for the paper: Gardens as science learning contexts across educational stages: learning assessment based on students' graphic representations

Examples of changes in paired graphic representations from pre-school, primary, secondary, and higher education

Pre-school Education

Low change

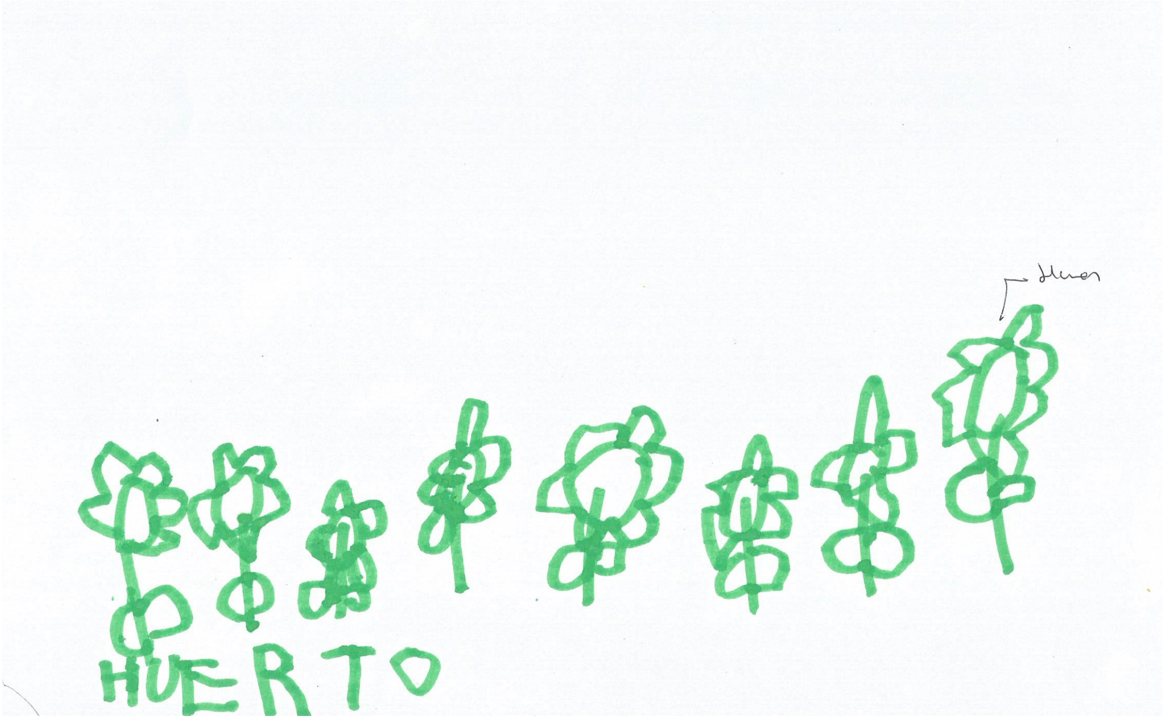

a) Initial

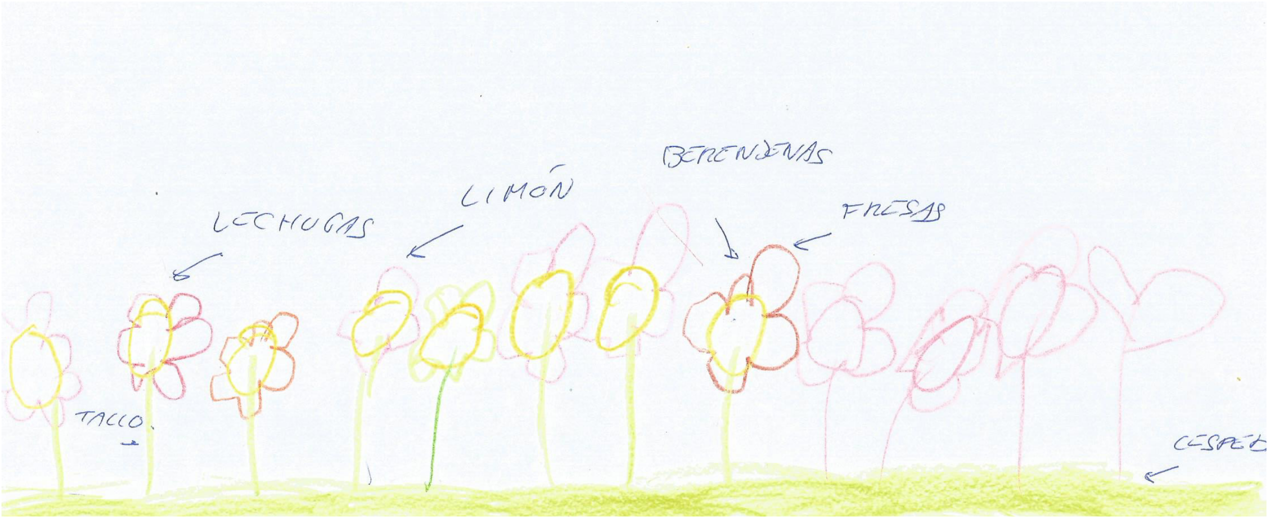

b) Final

Medium change

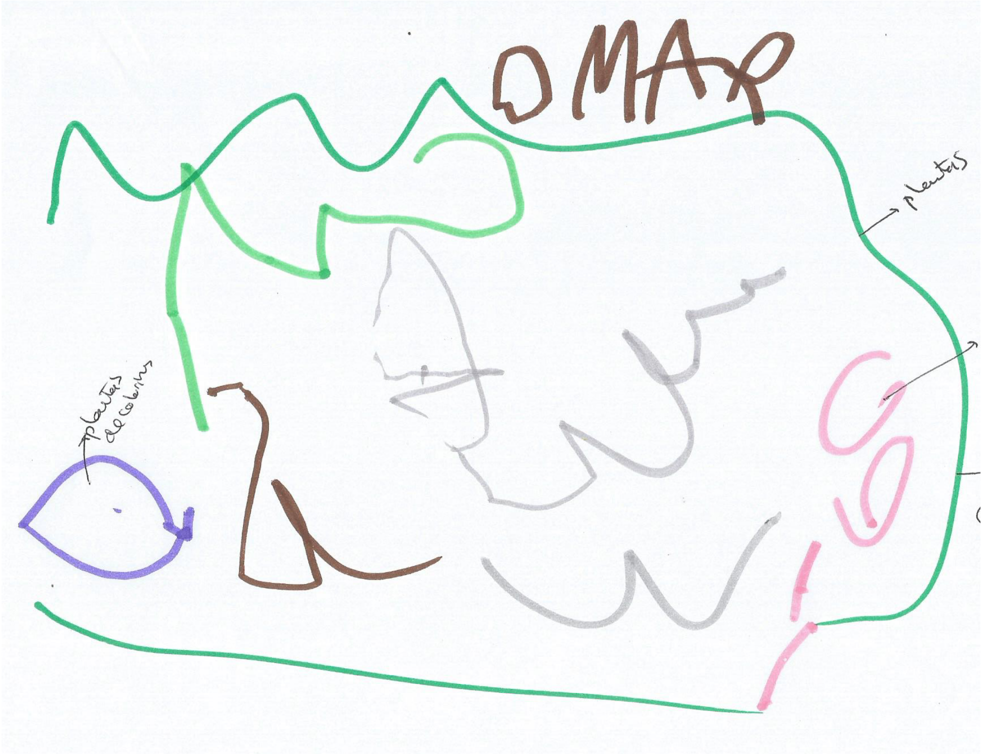

a) Initial

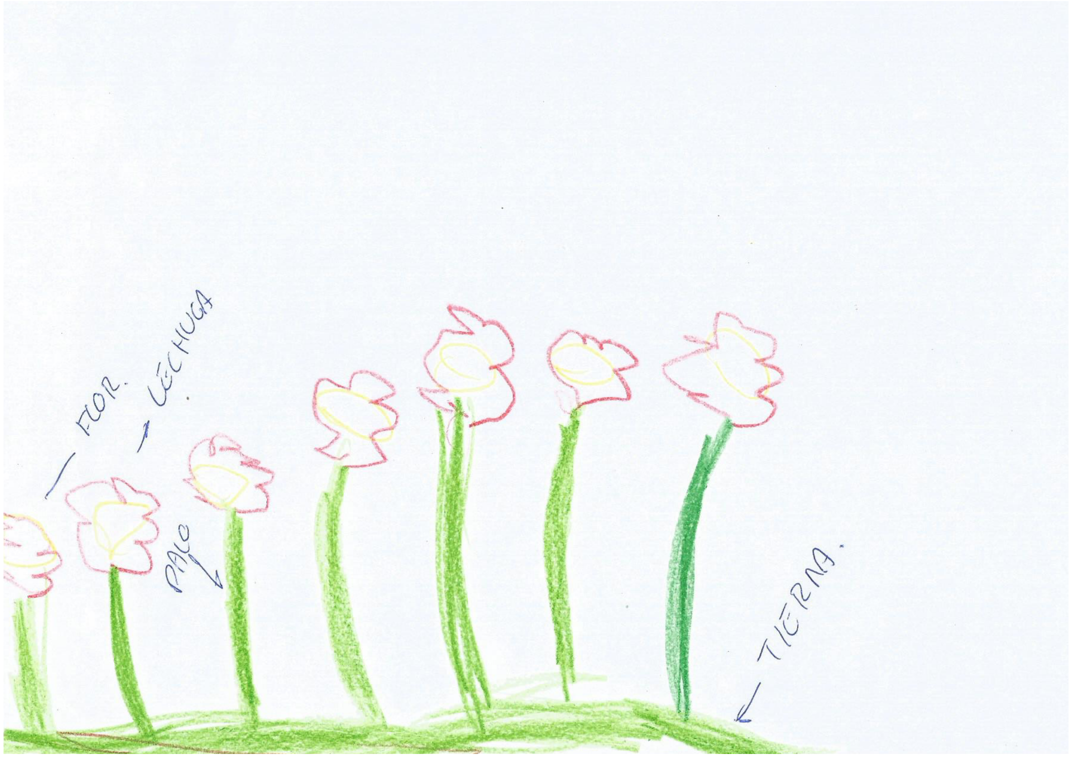

b) Final

High change

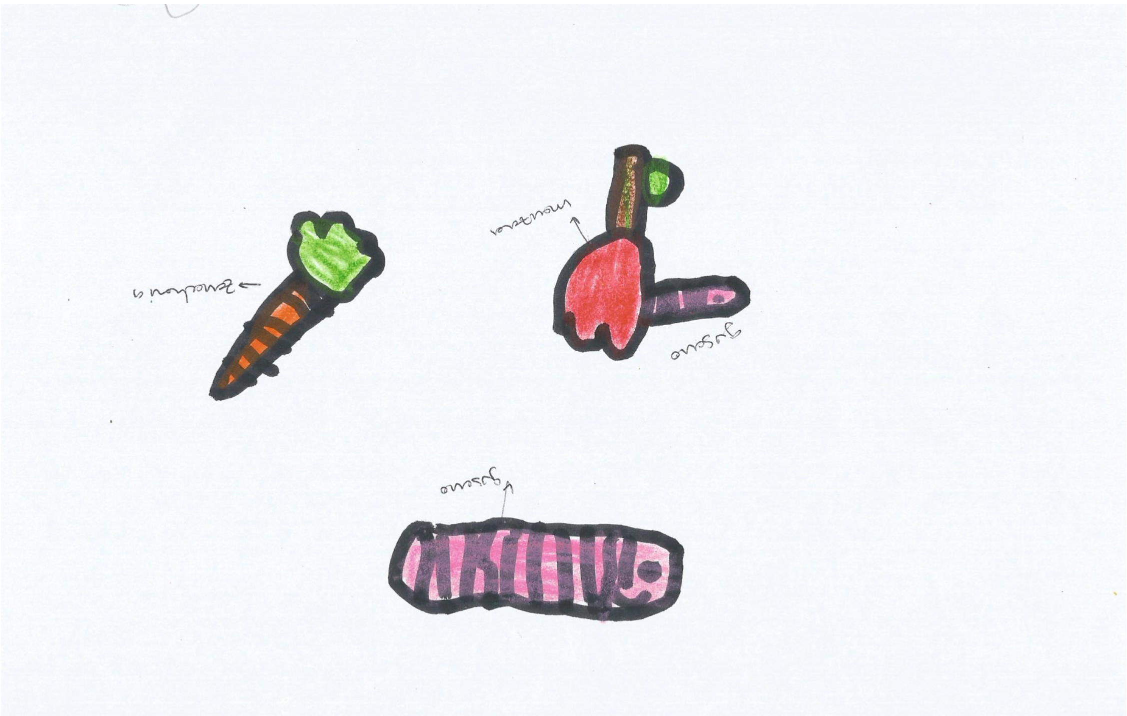

a) Initial

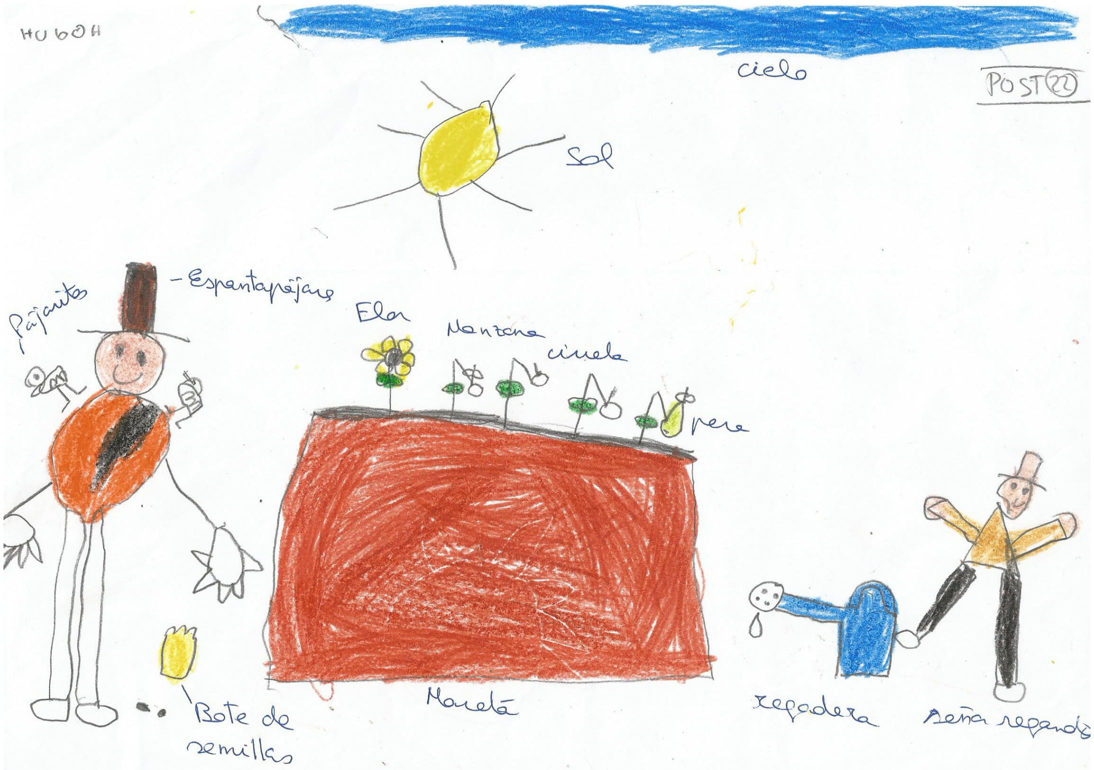

b) Final

Primary Education

Low change

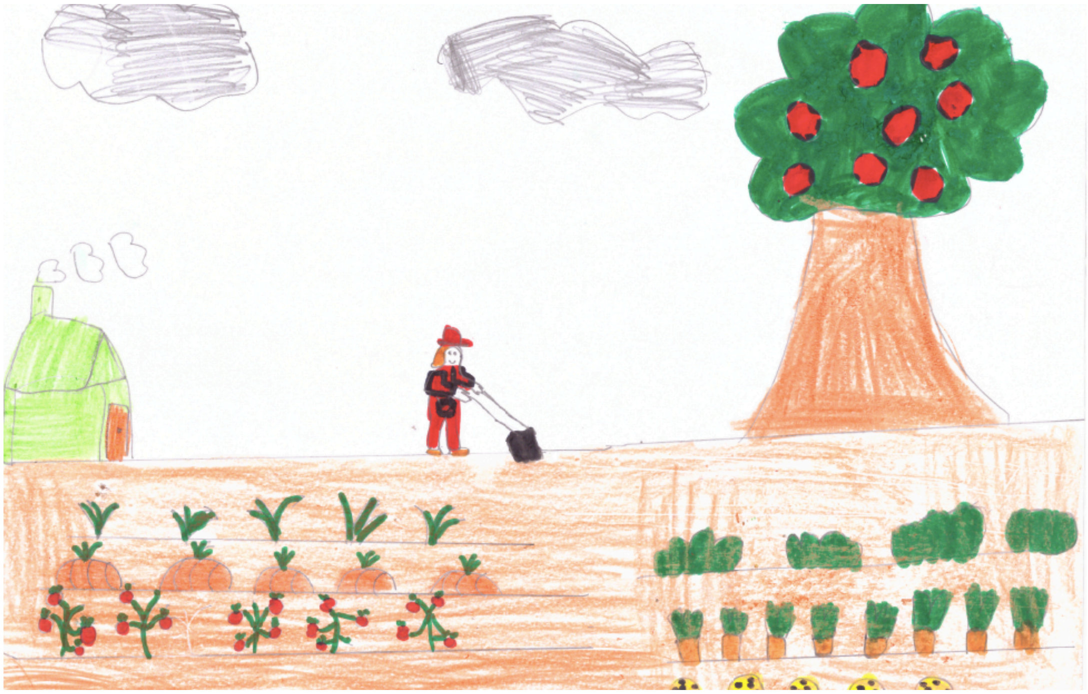

a) Initial

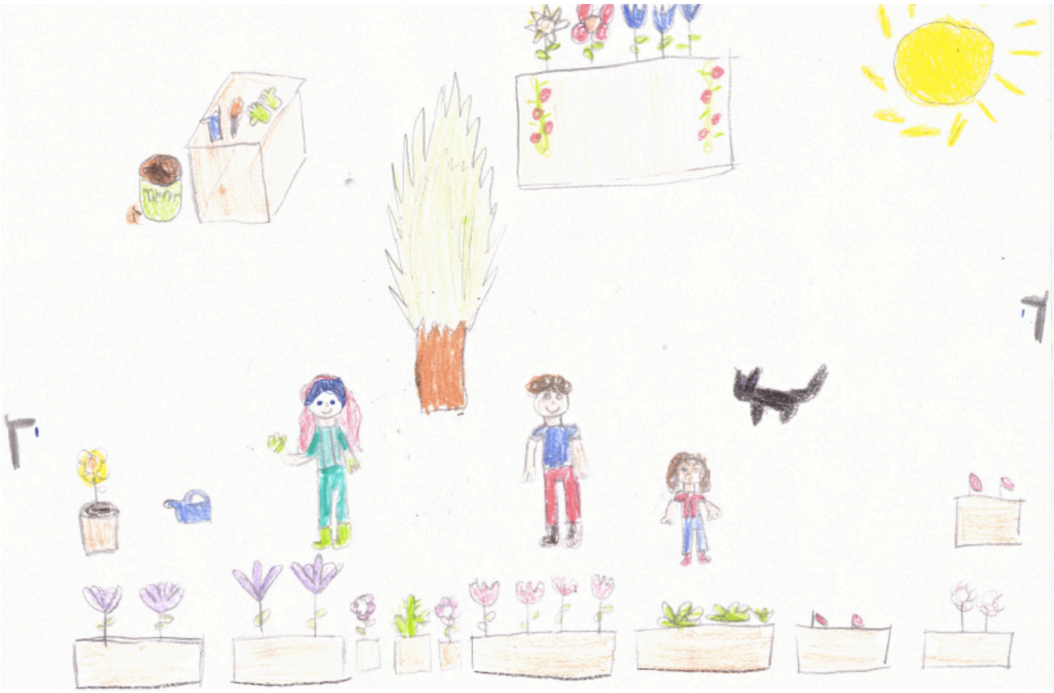

b) Final

Medium change

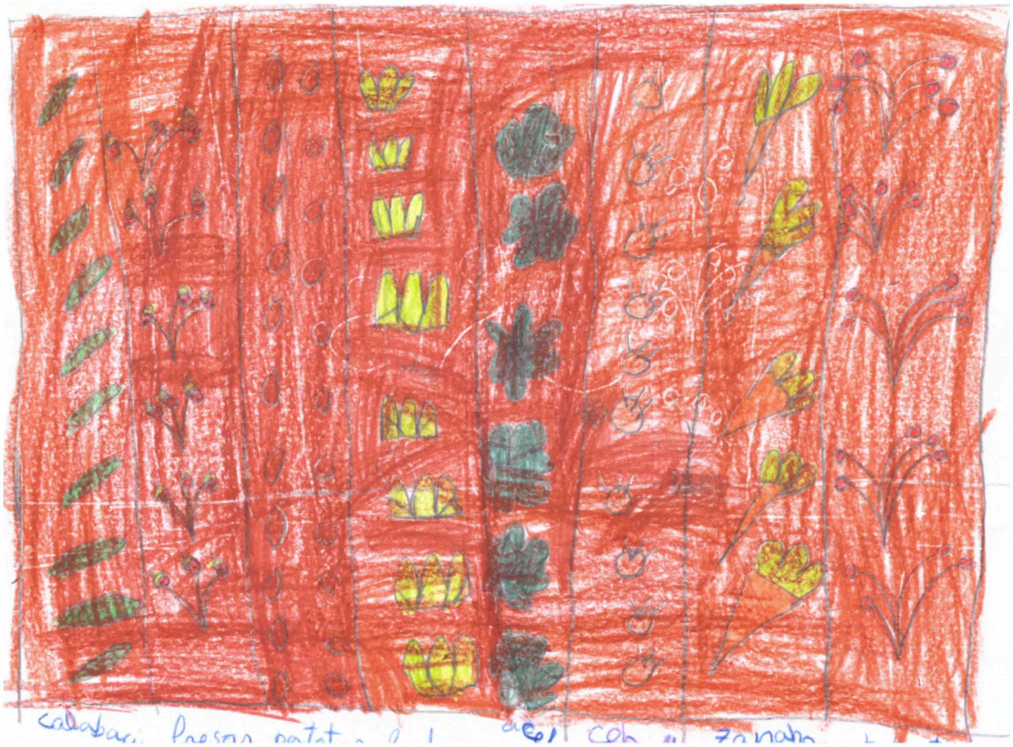

a) Initial

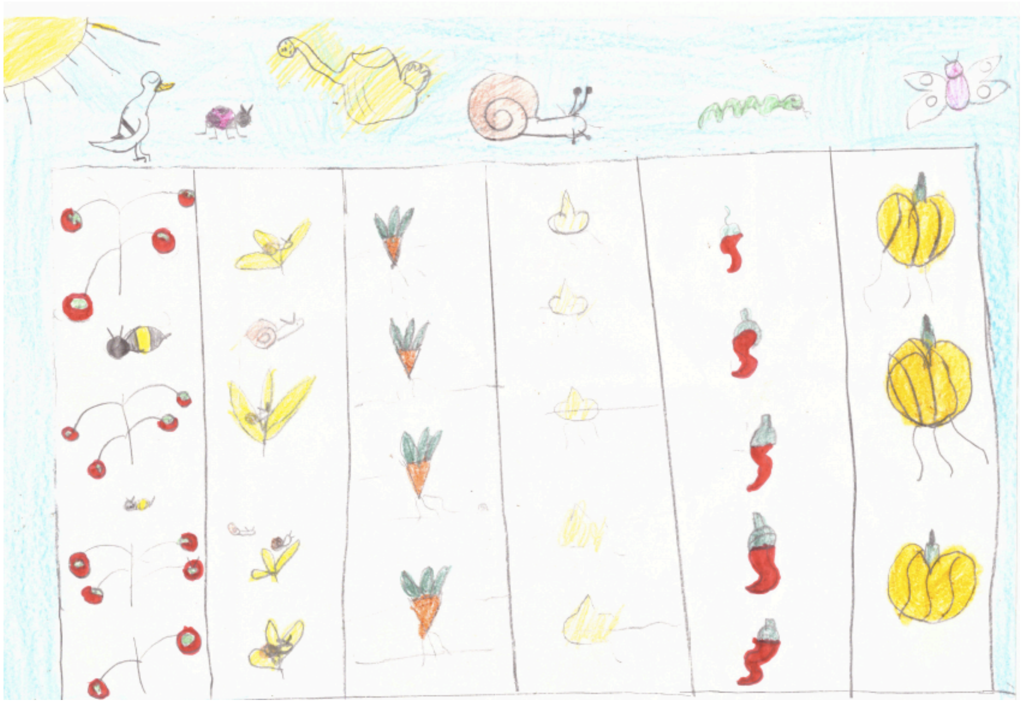

b) Final

High change

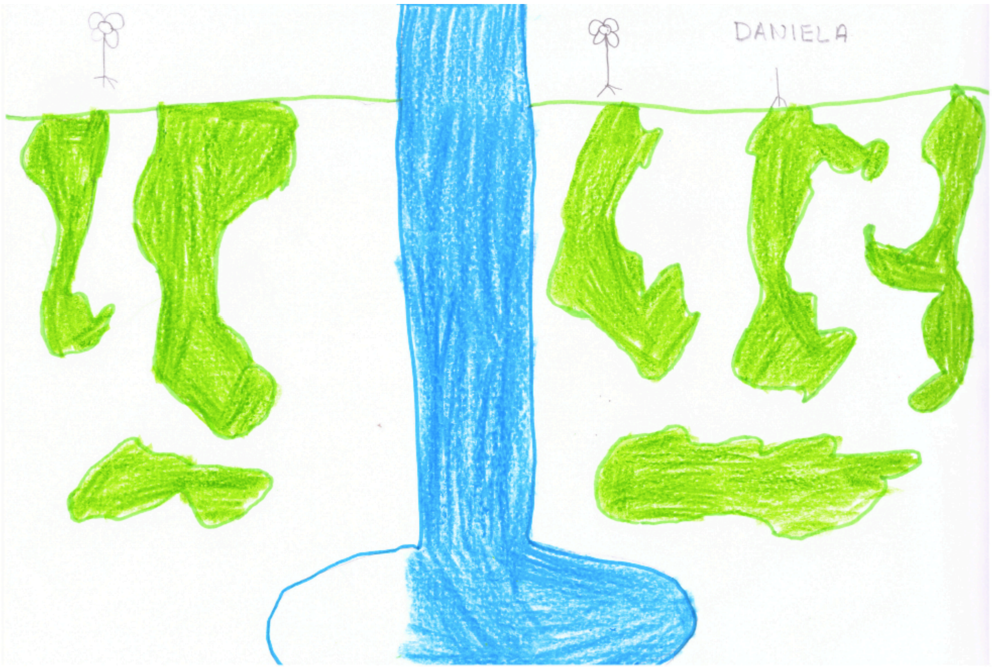

a) Initial

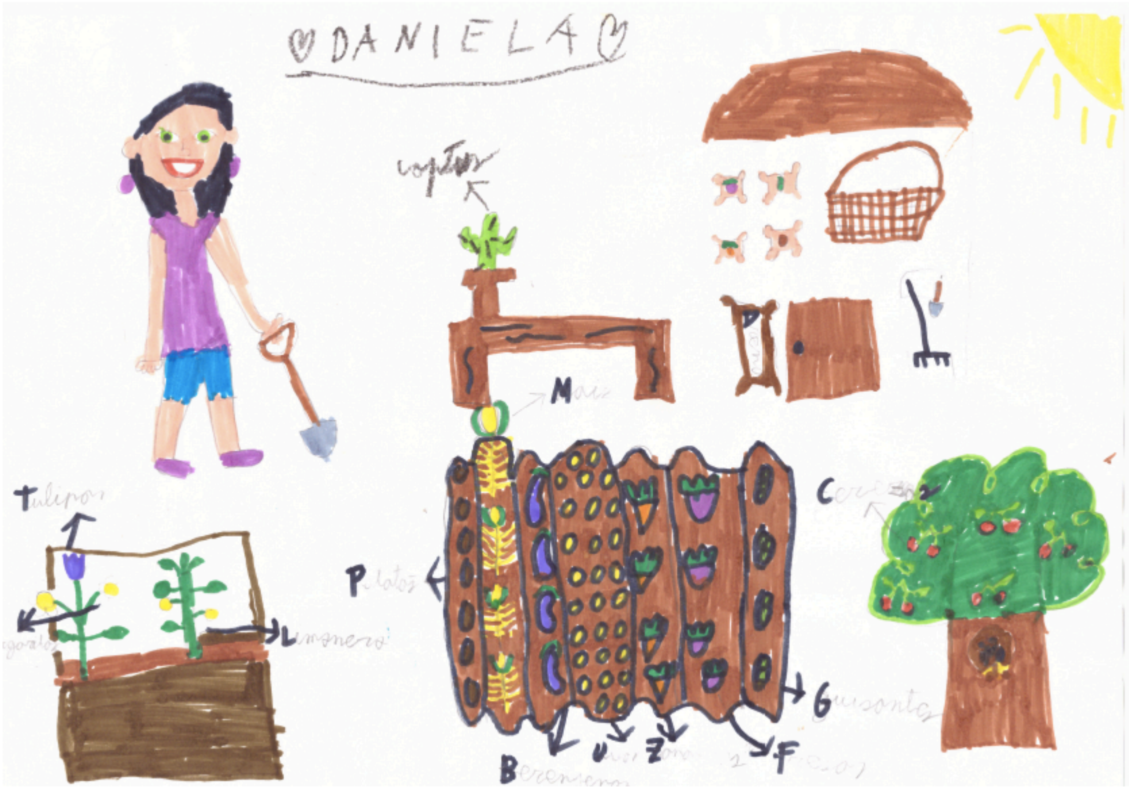

b) Final

Secondary Education

Low change

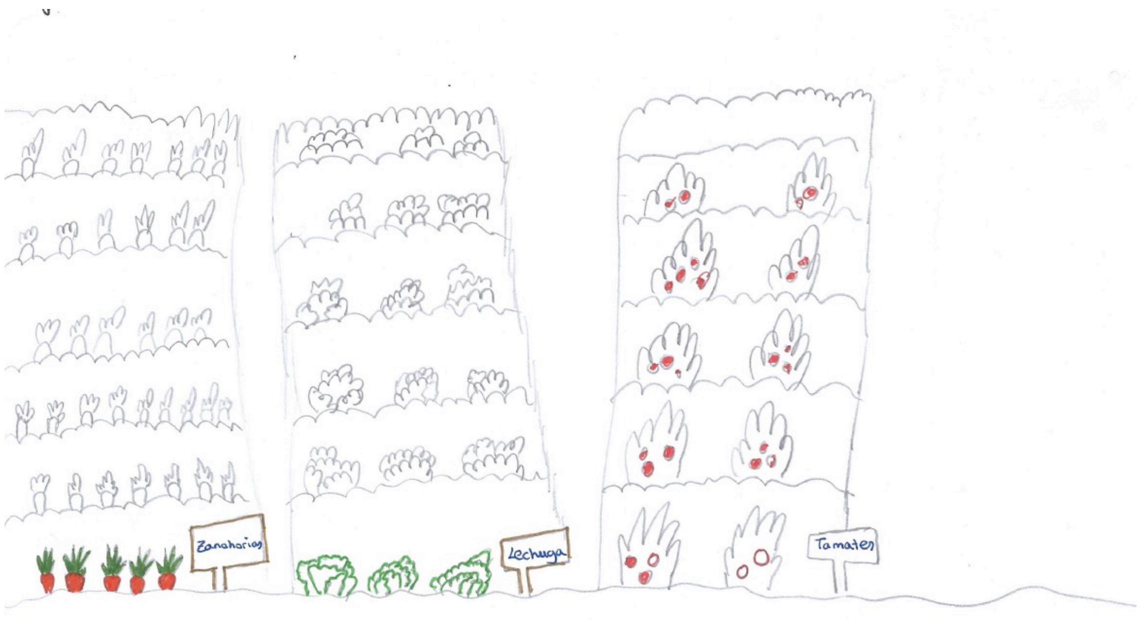

a) Initial

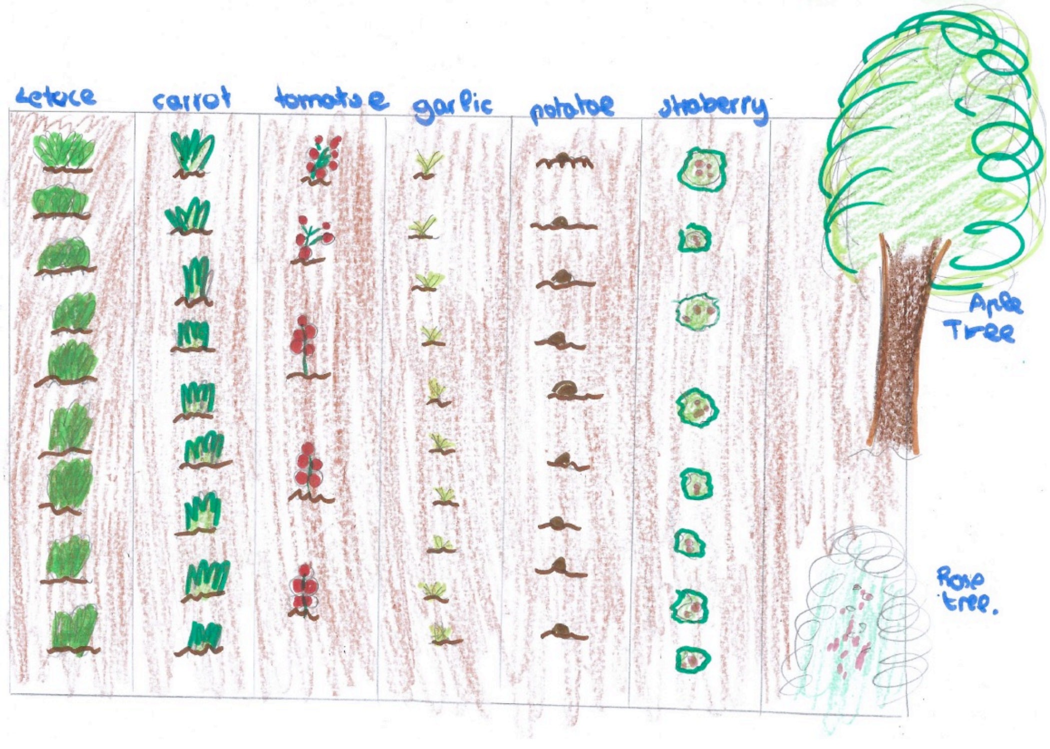

b) Final

Medium change

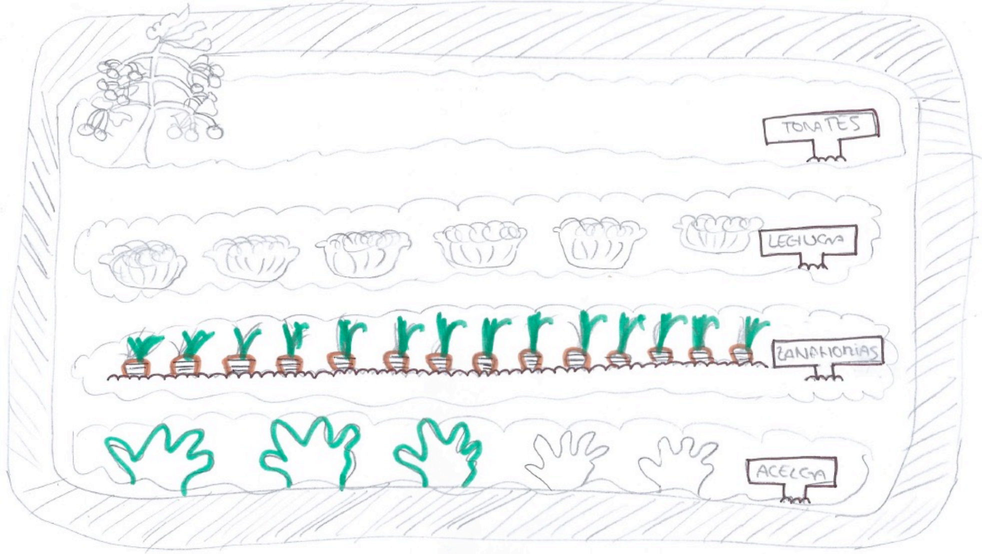

a) Initial

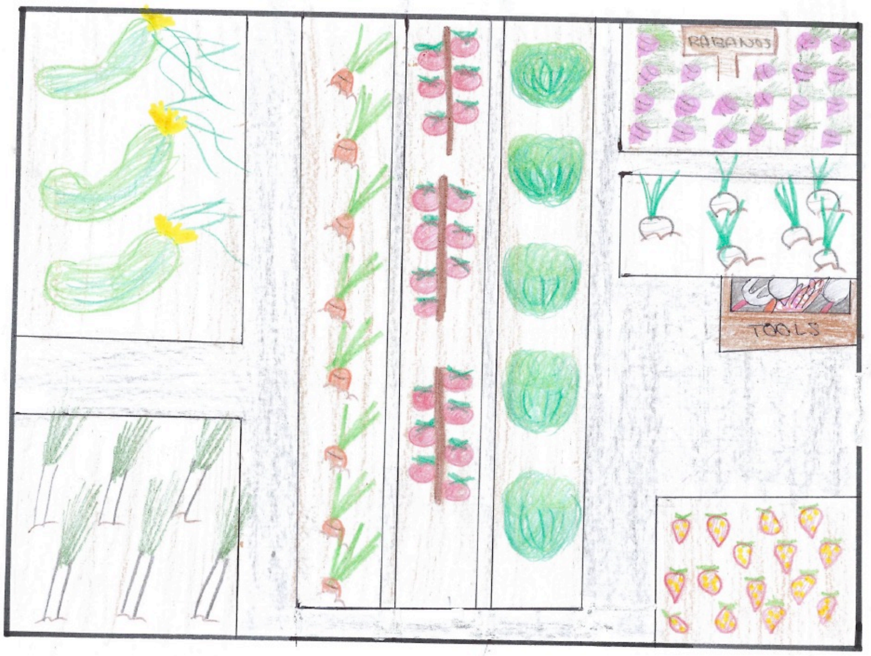

b) Final

High change

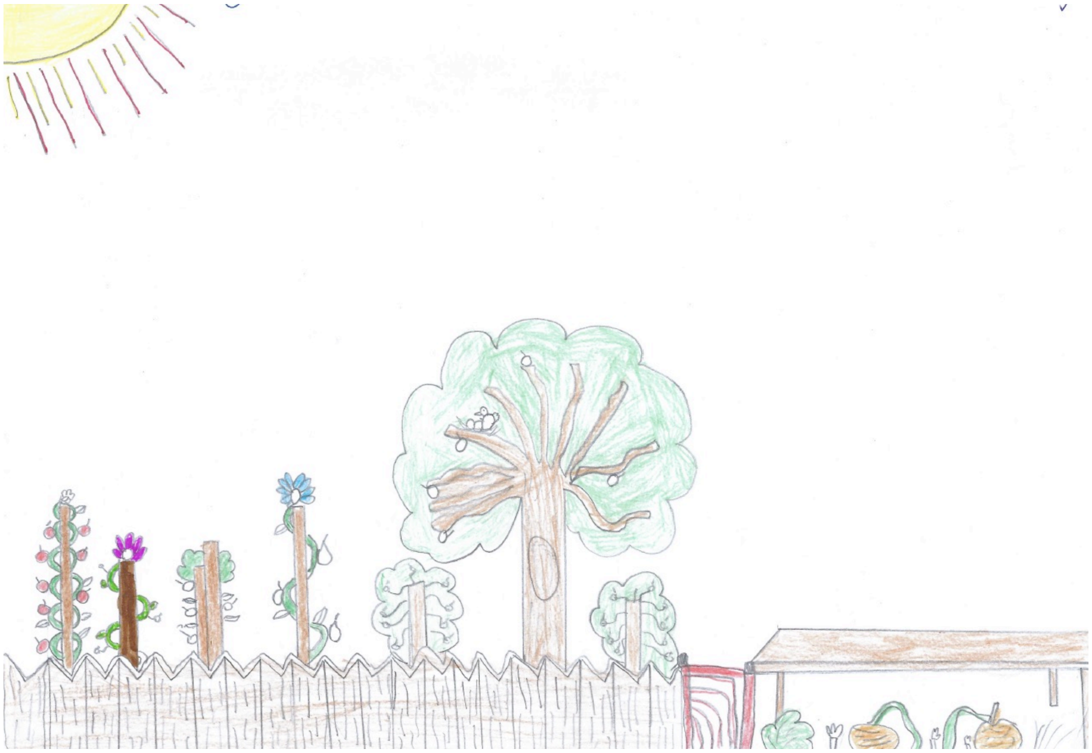

a) Initial

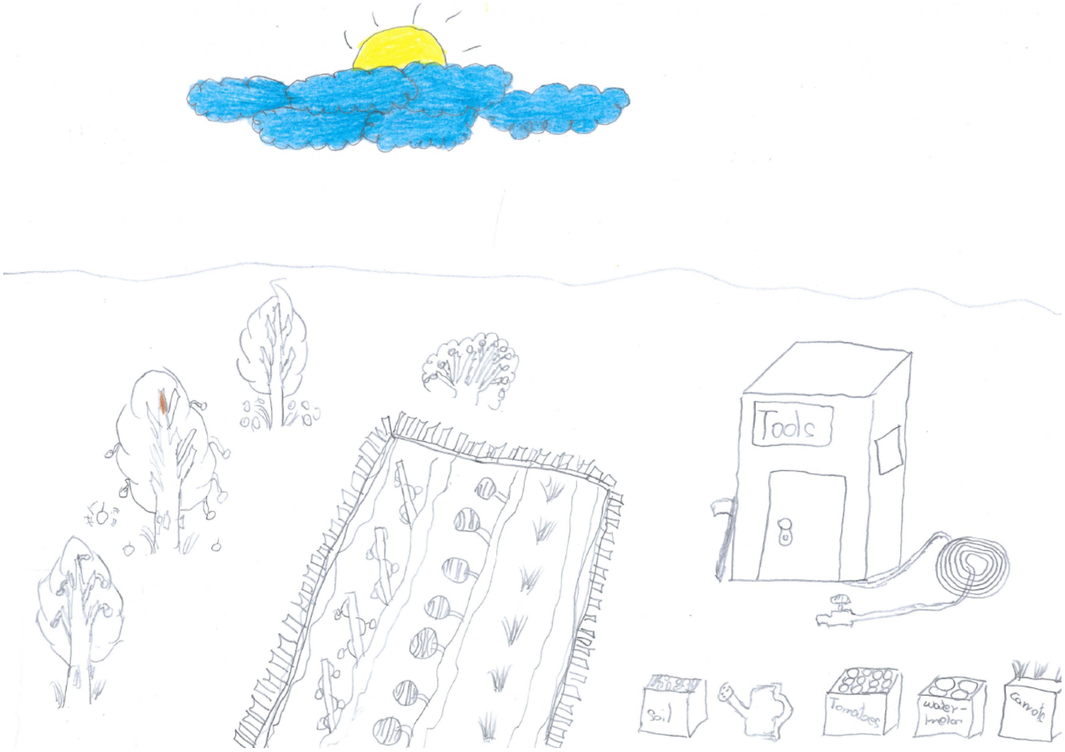

b) Final

Higher Education

Low change

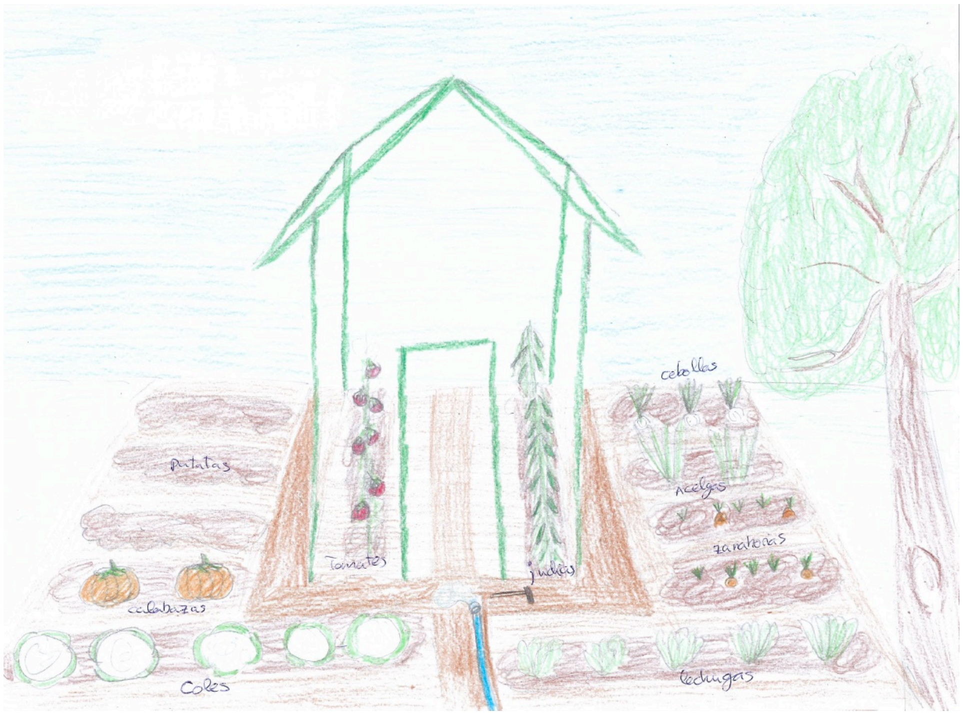

a) Initial

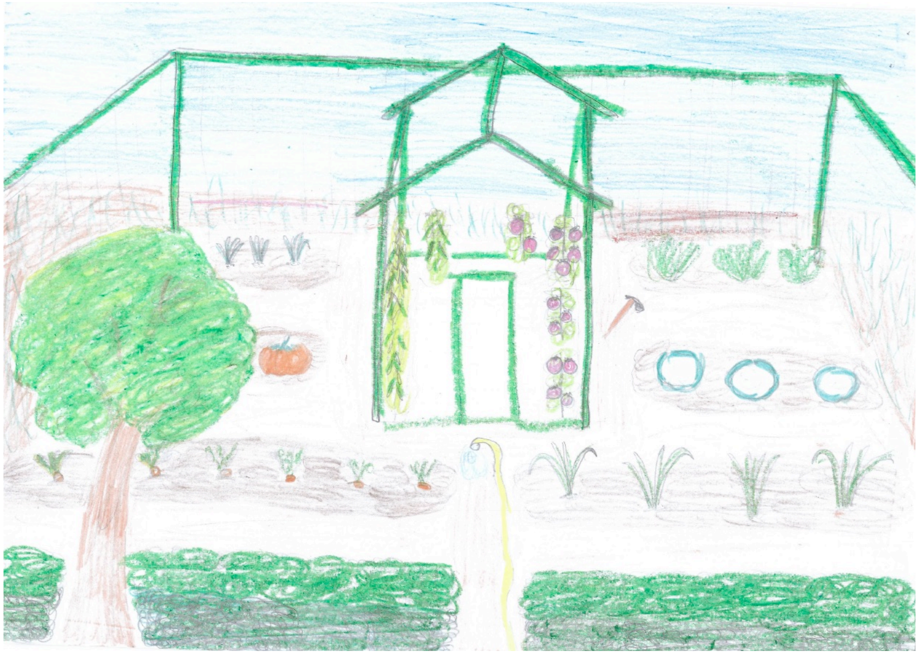

b) Final

Medium change

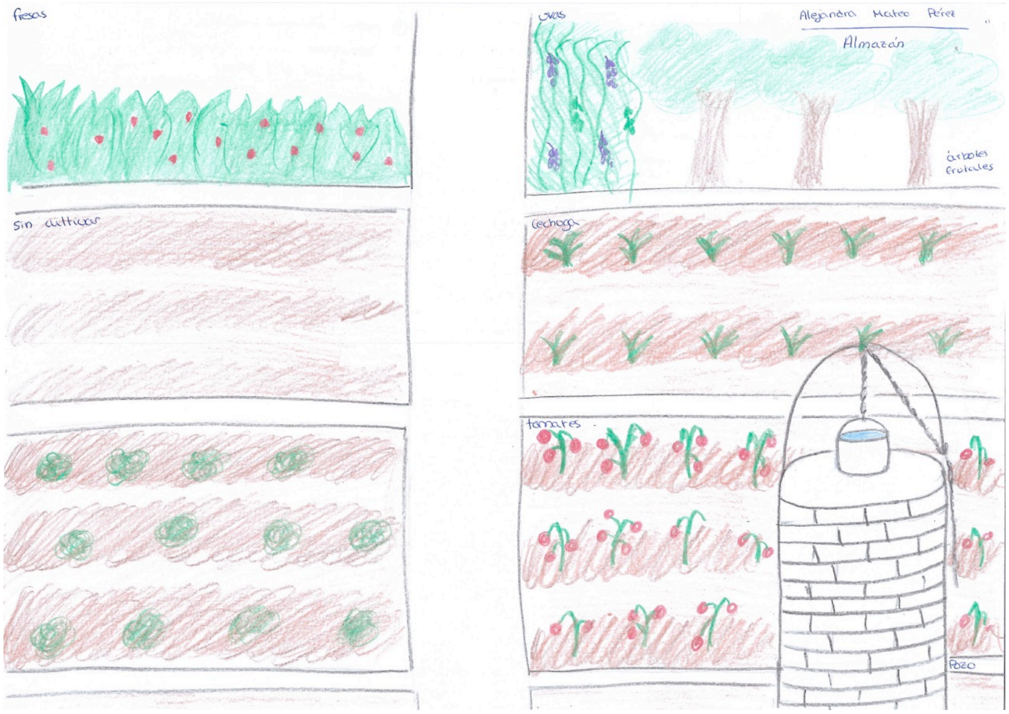

a) Initial

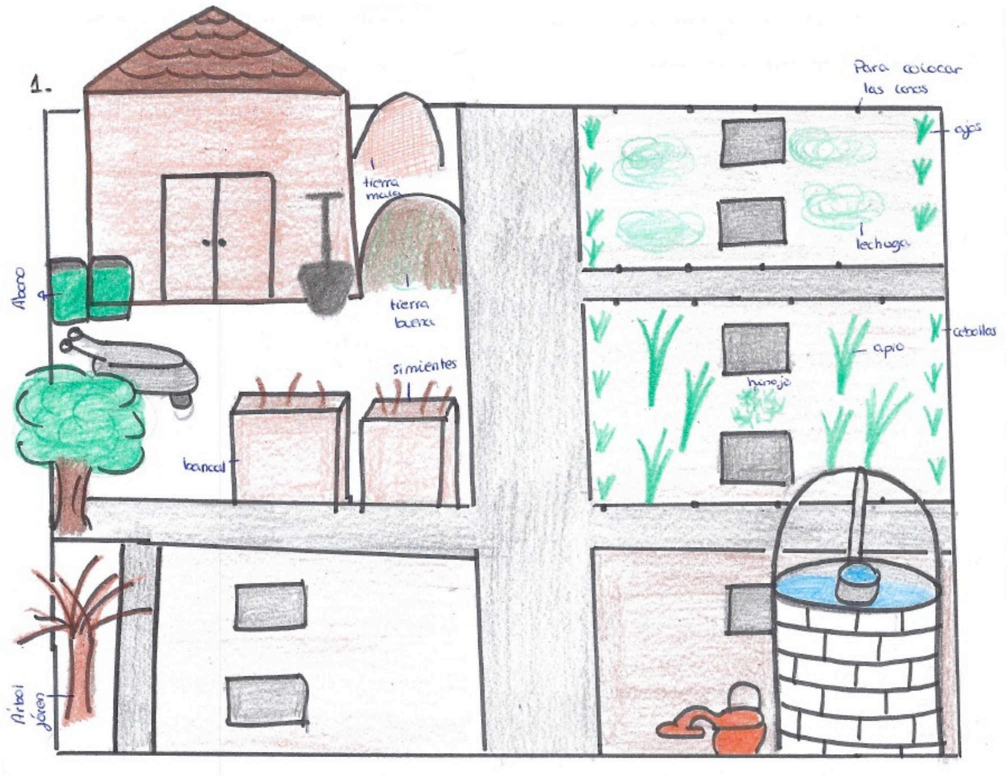

b) Final

High change

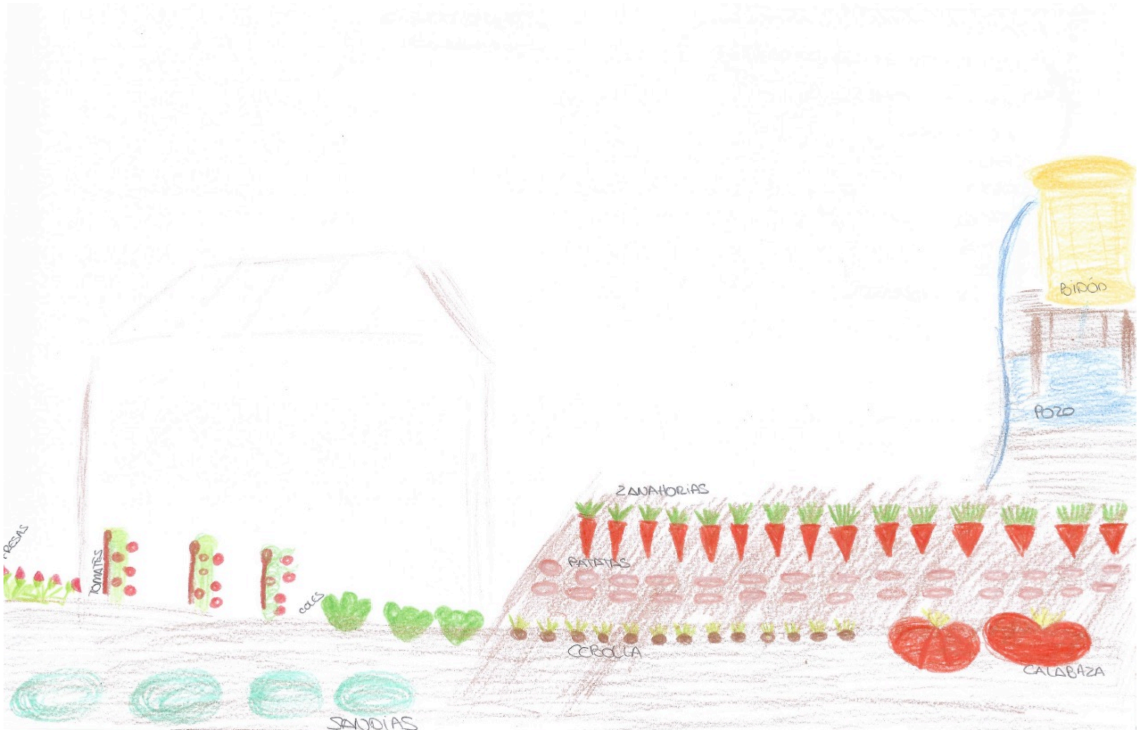

a) Initial

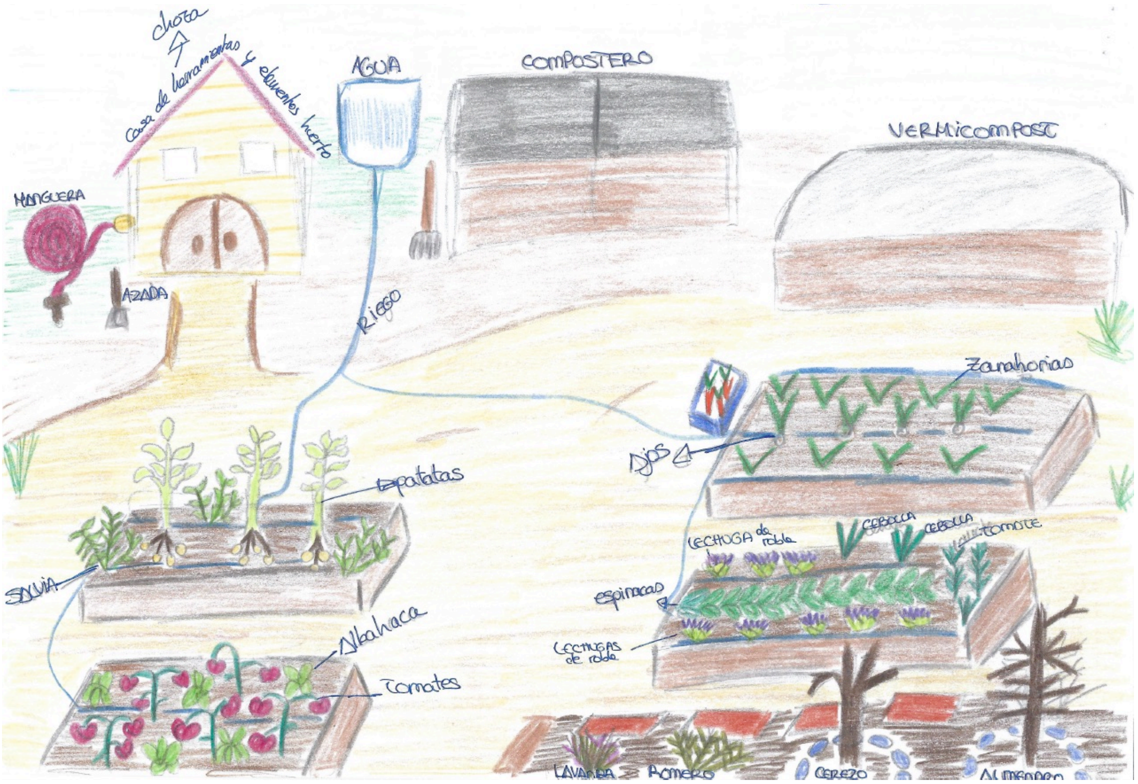

b) Final
